# From geology to biology: an interdisciplinary course in crystal growth

**DOI:** 10.1107/S1600576722008032

**Published:** 2022-10-01

**Authors:** Sergey G. Arkhipov, Tatyana B. Bekker, Anna A. Gaydamaka, Anna Y. Likhacheva, Evgeniy A. Losev, Elena V. Boldyreva

**Affiliations:** a Novosibirsk State University, Pirogova Street 2, Novosibirsk, 630090, Russian Federation; b Boreskov Institute of Catalysis SB RAS, Lavrentiev Avenue 5, Novosibirsk, 630090, Russian Federation; c State Research Center of Virology and Biotechnology VECTOR of the Federal Service for Surveillance in Consumer Rights Protection and Human Well-being (FSRI SRC VB VECTOR), Koltsovo, 630559, Russian Federation; d Sobolev Institute of Geology and Mineralogy SB RAS, Koptyug Avenue 5, Novosibirsk, 630090, Russian Federation; e Novosibirsk State University of Architecture, Design and Arts, Krasny Avenue 38, Novosibirsk, 630099 Russian Federation; Wilfrid Laurier University, Waterloo, Ontario, Canada

**Keywords:** crystal growth, polymorphism, high pressure, minerals, materials, teaching, biopolymers, pharmaceuticals

## Abstract

This contribution shares experience of teaching an interdisciplinary university course in crystal growth with examples ranging from geology to biology. This is an attempt to combine teaching the basics of the classical and non-classical theories of crystallization with impressive examples of crystals growing around us and in the human body, as well as demonstration of the common phenomena in the growth of minerals in nature, crystalline materials in industry and the laboratory, and biomimetic and stimulus-responsive crystals. Lectures are supported by laboratory exercises. Students can also perform an individual research project and present an oral contribution at a mini-conference. Examples of the topics considered in the course are given, and an extensive list of references to papers and web resources is provided, which may be useful to those who want to implement anything from the authors’ experience.

## Abstract 2

The authors share experience of teaching an interdisciplinary university course in crystal growth with examples ranging from geology to biology.

## 1. Introduction

Single crystals play a key role in the development of advanced technologies. All modern electronics, including wireless devices, contain single crystals of silicon as the main component. Not a single modern industrial technology can do without laser, acousto-optic, nonlinear optical, polarization and other single-crystal elements. The radiation of solid-state lasers, the working elements of which are commonly doped single crystals (yttrium aluminium garnet, Y_3_Al_5_O_18_, yttrium orthovanadate, YVO_4_, *etc*.), is characterized by exceptional coherence and monochromaticity. Fundamental research in elementary particle physics and the search for extremely rare events would be impossible without single-crystal scintillation detectors (Bi_3_Ge_4_O_12_, CdWO_4_, NaI:Tl, stilbene *etc.*) and cryogenic bolometers (Li_2_MoO_4_, Na_2_Mo_2_O_7_ *etc.*). Synchrotron and neutron studies are impossible without single crystals as well. The International Union of Crystallography pays much attention to the problems of crystal growth. The recent introduction of a special section in *Acta Crystallographica Section B*, in addition to the already available opportunities for publication of papers related to crystal growth in *Acta Crystallographica Section F* and *Journal of Applied Crystallography*, is a manifestation of this (Blake *et al.*, 2021 ▸).

Independently of its practical importance, crystal growth is a fascinating topic that always attracts people. Since ancient times, humans have admired crystals that have grown in nature as minerals. Observing crystals strongly influenced the development of science and culture (Garcia-Ruiz *et al.*, 2015 ▸). Crystals are visually attractive. Growing crystals is an experiment that can be performed easily in the household with cheap and accessible compounds like salt, sugar, water, naphthalene *etc*. (Wood, 1972 ▸; Jones, 1981*a* ▸,*b* ▸). Crystal growth competitions worldwide attract thousands of children [see https://www.iucr.org/outreach/crystal-growing-competition and Agbahoungbata *et al.* (2017 ▸), Boldyreva (2014 ▸), Lamas (2017*a* ▸,*b* ▸), Dacombe (2013 ▸, 2017 ▸), Ashcroft (2018 ▸) and Suescun (2014 ▸)]. Exhibitions of crystals – be they educational, focusing on artistic value, related to mythology and healing, or using crystals as jewelry – attract people everywhere. If a city has a mineralogical museum, it is always one of the major attractions for the general public and tourists, as well as for scientists representing various fields of research. Crystals can also be found in museums and at exhibitions dedicated to technology and engineering, with expositions illustrating how crystals are manufactured and how they are used (Garcia-Ruiz *et al.*, 2015 ▸; see also Appendix 1 in the supporting information).

Crystal growth remains to a large extent an art and requires experience and intuition. At the same time, it is based on a deep knowledge of physical chemistry (thermodynamics, kinetics), as well as of inorganic, organic, coordination and bioorganic chemistry (depending on which compound needs to be crystallized). The problems of crystal growth are closely related to solution chemistry, solid-state chemistry, mineralogy, geochemistry, materials science and, of course, crystallography.

This broadness at the same time accounts for the difficulties of teaching crystal growth. Currently, crystal growth is taught at different levels. First, there are many elementary practical courses, also available as videos and booklets, with children and the general public being the target audiences. Such courses give some basic ideas of how the crystals grow, but their main goal is to entertain and to provoke interest in this field. Second, there are special courses aimed at educating experts in crystal growth science and technology. Third, crystal growth is introduced within courses on crystal structure solution, since structural studies typically begin with the preparation of a crystal suitable for a diffraction experiment (Whelan *et al.*, 2018 ▸). Some facts about crystal growth can be also scattered through courses in other subjects: crystallization of minerals in nature is taught to earth scientists, protein crystal growth to life scientists and protein crystallographers, and crystallization of the compounds important for technical applications to materials scientists. These three approaches co-exist in parallel, usually without much overlap. Many textbooks and monographs are available (see Appendix 2 in the supporting information), but, to the best of our knowledge, there is not yet a book that integrates the basics and the applications in different fields. We attempted to develop a course that would combine the best practices from all three of the above-mentioned approaches: visual attractiveness, hands-on practices, introduction to the basics of crystal growth, and examples from different fields like planetary sciences, materials sciences and the life sciences. The course can be taught with moderate resources and can be modified flexibly, being adapted to the demands and backgrounds of the participants. In our course we use the experience of teaching crystal growth at various short-term international schools, such as an International Crystallization School in Granada (https://iscgranada.org/), having adapted this experience to the formal requirements of a long-term university course.

## 2. Teaching crystal growth at the Chair of Solid-State Chemistry of Novosibirsk State University

### 2.1. A general overview

At the Chair of Solid-State Chemistry of Novosibirsk State University we have experience of teaching crystal growth at different levels and to different audiences. We have taught crystal growth to children in a simple, attractive and entertaining way (Boldyreva, 2014 ▸; Gražulis *at al.*, 2015 ▸). We have also used the topic of crystal growth for public engagement, being heavily assisted by the lectures of our guests from all over the world (https://www.iucr.org/world/meeting-reports/russia/novosibirsk-school).

We give general lecture courses on solid-state chemistry (Boldyreva, 1993 ▸, 2010 ▸) and supramolecular chemistry (Varnek *et al.*, 2000 ▸) that include crystal growth as topics in relation to the synthesis, structure and properties of compounds and materials. Also, for the practicals in X-ray diffraction, the students need samples, and this is where they first encounter for themselves the problem of obtaining them.

Being inspired by the success of the public lectures, demonstrations and exhibitions dedicated to crystal growth on one side, and meeting the demand of students to assist them in obtaining the samples for practicals in X-ray diffraction on another, we have extended this activity, attempting to develop a course that would combine the attractiveness of a comprehensive description of the most interesting and sometimes unusual aspects of crystal growth in the world around us in nature and technology, an introduction into the basics of crystallization as a science, and the provision of some useful practical skills for growing a crystal when needed.

### 2.2. An interdisciplinary project-oriented course

This course intends to give impressive examples of crystals growing around us and in the human body, as well as to demonstrate what is common in the growth of minerals in nature and in the laboratory. We consider crystalline materials in industry and the laboratory, as well as biomimetic and stimulus-responsive crystals. Lectures are supported by laboratory exercises. Students can also perform an individual research project and present an oral contribution at a mini-conference. The course can be taken independently of other courses and is therefore self-sufficient. At the same time, many students combine this course with other courses that we give (Appendix 3), and benefit from the synergy.

The course lasts one semester and includes 18 lectures; one lecture lasts two academic hours, that is 90 min with a break. All students attend the lectures simultaneously. The number of those attending a lecture is not restricted, if space permits. However, only those students who have also received training in the laboratory can get credits for the course. If there is an urgent need for those who cannot be present in the audience during lectures, we have the technical option of attending on-line. Also, some lecturers (not from Novosibirsk, for example) teach on-line.

Laboratory practicals take 18 h, and self-study work dedicated to reading literature and preparing a mini-conference presentation also takes 18 h. Laboratory practicals include general instruction on safety and good practice in a chemistry laboratory, some demonstrations, some exercises that are performed by all the students, and one or more projects of choice on a more specialized topic. Some students spend much more time in the laboratory, since their main (bachelor, master, PhD) research project requires growing crystals. We support this additional work. Not more than ten people can be present in the laboratory simultaneously. If more students come to the course, we must divide them into two groups and provide laboratory work on different days, but this means extra work for us. Taking into account our limited space, staff and budget, our optimum number of students for a laboratory practical would be ten. Until now we have had up to 20 students to train, and this was rather difficult. Since the crystallization process can often take a long time (2–3 weeks), the samples are left in a special crystallization room and the students are encouraged to visit the laboratory at regular time intervals between the class hours, to observe how their crystals are growing. Laboratory exercises can be performed either individually or by a group of two students. The laboratory protocols describing all the details of performing each exercise, as well as a concise report on results, are evaluated by a tutor. After having read the written report, a tutor has an oral discussion with the student to check their understanding and to clarify the most important issues. At the end of the course we have a mini-conference at which all students present 10–15 min reports on a topic of their choice. These reports can be based on literature data, or on their own research project that was carried out either using the equipment of Novosibirsk State University or at one of the Institutes of the Siberian Branch of the Russian Academy of Sciences. The final evaluation of a student is based on their presentation at the mini-conference and the results of the laboratory exercises and their interpretation.

A general overview of the course is given in Table 1 ▸. Table 2 ▸ gives some examples of topics of laboratory exercises and research mini-projects are given. Examples of the titles of student presentations at the final student mini-conferences are given in Table 3 ▸.

#### 2.2.1. Lectures

We start with a general introductory lecture giving an overview of crystal growth in nature and techniques. We use photographs and videos showing crystals and the processes of their growth [both from the web (see links in Appendix 4) and our own]. We also bring sample crystals, grown by ourselves. In principle, it is possible to borrow some crystals from a mineralogical museum. A dialog with the students helps them to share their own experience of observing, growing and using crystals. We also discuss where crystals can be present in the classroom (metals, chalk, pencils, computers, smartphones, clothes, lamps, chocolate *etc*.). We also try to find crystals in the human body – in a young and healthy one and in that of a sick and/or aged person. At this stage we also discuss crystals present in food. A tentative plan of this introductory class is given in Appendix 5a.

The first block of five lectures gives a basic introduction to the thermodynamics and kinetics of crystallization, the classical and non-classical theories of crystallization, phase diagrams and their role in the control of crystallization, and the role of impurities, surfactants and various types of defects. We consider what defines the equilibrium and the non-equilibrium shapes of crystals, the problems of polymorphism control, disappearing polymorphs, and quenching and annealing of metastable phases. This part of the course is probably the most traditional one and is well supported by many textbooks and monographs, as well as by review papers. Examples of the sources we use are given in Appendix 2, although other sources can be used, depending on the preferences of the teachers and the language of instruction or the native language of the students. As an illustration, a tentative plan of one of the lectures from this block is given in Appendix 5b.

We proceed with other phenomena that are related to crystal growth on Earth and on other planets. The processes of mineral formation from the melt, from solution, from vapor, and at high pressures and temperatures are discussed in the context of geochemical and geological phenomena. This block of four lectures is concluded by a visit to the Mineralogical Museum of the Sobolev Institute of Geology and Mineralogy Siberian Branch of Russian Academy of Sciences, and this visit is one of the highlights of the course. We also suggest as an option an excursion to an enterprise where artificial minerals (emeralds, diamonds and others) are grown at high pressures and temperatures. Mineralogical museums are available in many places (see Appendix 1). Therefore, in other cities and countries a teacher can arrange to visit another museum. If this is not possible, virtual on-line museum visits are nowadays possible. Also a mineralogical excursion in the neighborhood, exploring sand, rocks, pebbles *etc*., is always an option. As an example, a tentative plan of a lecture from this block is given in Appendix 5c.

The next block includes four lectures on various topics which illustrate complex phenomena that can be observed on crystallization, and some crystallization techniques that are not so common (like high-pressure crystallization, crystallization in confined media, racemization and chiral resolution on crystallization, and mechanical phenomena accompanying crystallization).

We have selected these topics since they are cross-disciplinary, related to problems of planetary sciences, life sciences, chemical engineering, materials sciences and supramolecular devices, and allow one to illustrate the similarities of phenomena that are observed on crystallization of minerals and both inorganic and organic compounds. These are also the topics of our own research, and therefore we can use many examples from our own experience, in addition to borrowing case studies from the literature. As an example, a tentative plan for a lecture from this block, one on high-pressure crystallization, is given in Appendix 5d. The literature sources in Appendix 2 can be used to prepare lectures on the above-mentioned topics.

The last block includes three lectures related to life and pharmaceutical sciences, as well to the food industry and biomimetic materials. We illustrate the possibility and the importance of the problem of biomineralization, including crystal growth in the tissues and cells of living bodies. Samples that the students have seen when visiting a mineralogical museum, and photographs available from different web resources (Appendix 4) and from our own practice, can be used as illustrations. Crystals in food (ice cream, chocolate, honey) are also considered, and this is always one of the most delicious topics. To make a lecture more memorable one can suggest to taste pieces of chocolates of different types and compare the impressions. One can also give small portions of ice cream that have been frozen differently and differ in the softness of taste because of the difference in the size and shape of the ice crystals inside. These ‘educational tools’ are used at the Granada crystallization school (https://iscgranada.org/), and this can work perfectly well in a regular university course, if resources and regulations (*e.g.* food in a lecture hall) permit. Students can be also instructed to carry out these exercises at home and report the results in the class. An important part of the course is an introduction to protein crystallization. These lectures give a general overview of this field, often raising significant interest. This is especially true in recent years, when the pandemic demonstrated the impact of modern crystallography on biology and on the development of drugs and vaccines, and the importance of this research direction for society in general. This part serves as a good introduction to another forthcoming course in protein crystallography for those who decide to seek deeper training in this field. A plan for a lecture in this direction is given as an example in Appendix 5e. Also, publications listed in Appendix 2 can serve as a source for many ideas.

#### 2.2.2. Laboratory exercises

The lectures are supported by practicals – hands-on work in the laboratory (Table 2 ▸).

Before starting any work in the laboratory, students are instructed about general chemical laboratory safety, electro-technical safety, fire safety, and safety related to using glassware and sealed ampules under pressure. Those students who continue a research project in a research laboratory also get specific safety instructions relevant for the selected work in the selected laboratory. The compounds and solvents for the compulsory part 1 of the laboratory exercises are selected to minimize the use of toxic, flammable and explosive substances, and can be used without special precautions, so that it is possible to use a laboratory bench without explicit need for a fume hood.

The laboratory exercises can be divided into two blocks.

The first block is the same for everybody and includes exercises that illustrate the basics of crystallization. These experiments do not require expensive reagents or sophisticated equipment and can therefore be performed by all the students. Most of these experiments follow the protocols that are described in textbooks. Some references are given in Appendix 2.

Crystallization of alums by slow evaporation of an aqueous solution using a seed aims to obtain as perfect a crystal as possible, to analyze its shape or to obtain multi-layer multi-color crystals alternating the solutions of alums of different ions. After the crystals have been grown, the students may keep them as a souvenir. They can also put the crystals on a single-crystal diffractometer, to index crystal faces and relate the equilibrium crystal shape with bulk crystal structure. Students are encouraged to make a sphere out of a crystal and to put the crystal again into a thermostated supersaturated solution. They can then watch how the faces develop. This exercise is described as an example in Appendix 6a.

Crystallization of NaCl in pure water and in the presence of impurities illustrates the effect of surface interactions on the crystal shape without changing the inner crystal structure.

Crystallization of glycine in the presence of different impurities illustrates the possibility to control both polymorphism, *i.e.* the bulk crystal structure, and crystal habit. A recent review (Boldyreva, 2021 ▸) contains references to the original publications from which the examples of protocols for this exercise can be taken.

The aim of the next exercise is to illustrate that there are multicomponent crystals like co-crystals, molecular salts and ionic co-crystals, and to discuss the structures of these types of solids. This exercise is based on the slow evaporation of a solution from a drop, which is placed on a glass with a modified hydro­phobic surface (Rychkov *et al.*, 2014 ▸). The crystals can be separated easily from this surface. It is straightforward to observe the crystal growth in a drop using an optical microscope. In some cases, well faceted crystals can be obtained. In many cases, especially for organic compounds, this method can be used as one of the most convenient methods of obtaining crystals suitable for X-ray diffraction. This exercise is described as an example in Appendix 6b.

A special topic is the effect of crystallization conditions on nucleation and nucleus growth. The aim of this exercise is to compare the polymorphs that crystallize (i) on antisolvent crystallization when different antisolvents are used, (ii) on slow evaporation and (iii) on spray-drying. This exercise is usually done using glycine as a case study, on the basis of our own research experience (Losev *et al.*, 2013 ▸). One can also use other compounds, depending on the availability of reagents and the interests of the students. The products of crystallization are compared using X-ray powder and single-crystal diffraction and IR and Raman spectroscopy.

The next two exercises of this block are dedicated to crystallization from the gas phase and to the crystallization of poorly soluble compounds. Crystallization from the gas phase can be demonstrated in this block by simple textbook examples of naphthalene or benzoic acid. More sophisticated experiments aiming to obtain practically interesting inorganic (Sankar *et al.*, 2002 ▸) and organic (Ye *et al.*, 2018 ▸; Guo *et al.*, 2020 ▸) compounds can be optionally performed when working on an individual project in block 2 of the practicals with more sophisticated equipment.

When studying crystallization of multi-component salts/co-crystals, which can be formed by mixing solutions of the components, students compare the outcome of fast mixing of two solutions and of a slow counter-diffusion. The difference in the particle size – fine powder versus single crystals visible with the eye – is obvious. This work is done in the ‘compulsory block’ using crystallization of CaCO_3_. Additionally, the data obtained by IR spectroscopy, X-ray diffraction and thermal analysis are compared to test if the crystal structures are the same. This topic can be further explored in a research project in part 2 of the practicals. The mini-projects can be dedicated to compounds like PbC_2_O_4_ (Virovets *et al.*, 1993 ▸) or Ni di­methyl­glyoximate (Bruce-Smith *et al.*, 2014 ▸), which give the same polymorphs, independent of the crystallization method, or to some organic compounds where the resulting polymorph depends on the crystallization protocol. An example where the obtained polymorph depends on the crystallization rate is the ‘glycine–oxalic acid–water–methanol’ system (Tumanov *et al.*, 2010 ▸; Losev *et al.*, 2013 ▸).

The last laboratory work from the ‘compulsory’ block is dedicated to the crystallization of proteins using the simplest case of lysozyme as a case study (Appendix 6c). The crystallization protocol was taken from Klinke *et al.* (2019 ▸, Appendix S1). The material for the crystallization can be purchased from *e.g.* PanReac Applichem. Optionally, for those who are especially interested in the crystallization of biological objects, we suggest going through all the stages of obtaining lysozyme starting from chicken egg protein according to the procedure described by Kuchkaev *et al.* (1996 ▸). When preparing this exercise one can also be inspired and instructed by the experience shared by Luft *et al.* (2010 ▸).

The second block of laboratory exercises is dedicated to individual projects, which are selected by the students depending on their interests. The results of this work can be reported at a mini-conference, or in the form of a literature survey and report. The topics of these projects can be suggested by the students themselves. Some of these projects cannot be performed in the university laboratory room and are carried out in a specialized research laboratory under the supervision of an expert. This holds, for example, for high-pressure high-temperature experiments, freeze-drying, electrocrystallization, microspacing in-air sublimation growth, or crystallization of proteins more complex and expensive than lysozyme. We cannot afford to let every student operate a diamond-anvil cell or a large-volume press or freeze-drying instrumentation. However, selected students can be trained individually to use this expensive equipment and more expensive reagents, if they are very motivated and interested. Some students use the project performed during our course as a part of their research qualification (bachelor, master, PhD) project, benefitting from our facilities and expertise. The examples for the second optional block are usually taken from our own research experience or from the original scientific literature. References are given in Appendix 2. For example, an exercise based on the experiments described by Chupakhin *et al.* (1987 ▸) can illustrate how the presence and concentration of defects can influence the mechanical properties of the solid. The mechanical strength of NaCl whiskers is shown to be significantly higher than that of ‘common’ crystals containing dislocations. The relation of the microhardness of NH_4_Cl to the concentration of Cu^2+^ added as an impurity on crystallization is illustrated in another set of experiments. An unusual seeding effect on high-pressure crystallization is illustrated on the basis of work reported by Zakharov *et al.* (2016 ▸).

When necessary, crystallization experiments are complemented by characterizing the products using optical microscopy, IR and Raman spectroscopy, and X-ray powder or single-crystal diffraction. When appropriate, for example, when comparing the polymorphs of organic compounds, the samples are also characterized by differential scanning calorimetry.

**Figure 1:**
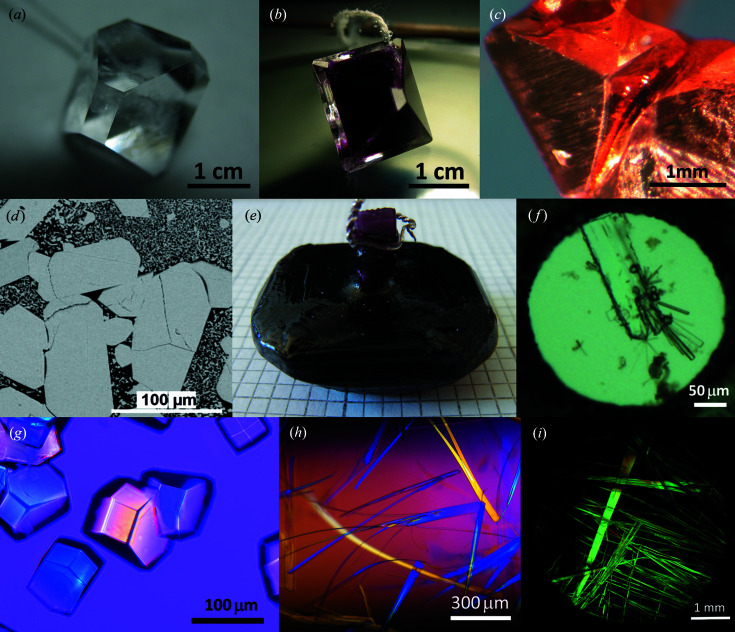
Examples of crystals grown by different techniques: (*a*) potassium alum crystals grown by slow evaporation from aqueous solution using seed crystals; (*b*) a layered alum crystal [chrome alum core (inside) covered by potassium alum shell (outside)]; (*c*) a copper crystal grown by redox reaction between CuSO_4_ and Fe; (*d*) a high-pressure high-temperature modification of BaB_4_O_7_ synthesized using the ‘Discoverer-1500’ at 3 GPa, 1273 K; (*e*) an NaBa_12_(BO_3_)_7_F_4_ crystal grown from a high-temperature solution by top-seeded solution growth; (*f*) recrystallization of β-chlorpropamide (prismatic crystal) into γ-chlorpropamide (needles) in a diamond anvil cell at 0.1 GPa in a pentane–iso­pentane (1:1) mixture; (*g*) lysozyme crystals crystallized by the hanging-drop vapor diffusion antisolvent technique (viewed in polarized light); (*h*) plastic l-isoleucine single crystals grown inside a glass capillary by layering diffuse antisolvent crystallization (viewed in polarized light); (*i*) copper chloride dihydrate crystals grown by slow evaporation from a drop (viewed in polarized light). Photographs were taken by Dr E. Losev (*a*)–(*c*), (*g*)–(*i*), Dr Sci. T. Bekker (*d*), (*e*) and Dr Sci. B. Zakharov (*f*).

Examples of the samples grown by students during laboratory exercises and research mini-projects are given in Fig. 1 ▸ and in Appendix 7.

#### 2.2.3. Mini-conference

The course ends with a mini-conference at which students can present the results of their literature reviews (a journal-club format) and the results of their own practical work on a selected topic. The topics for the literature reviews are suggested by the teachers, but can also be proposed by the students themselves. Examples of the topics that were selected for last year’s mini-conference are listed in Table 3 ▸. Interestingly, and contrary to our expectations for a mini-conference, the students often tended to select a topic different from their background at the university, *i.e.* geology students often take topics related to life sciences and *vice versa*, biologists or organic chemists take topics related to crystallization of minerals and geochemistry. This suggests that our intention to give the students a broad integrating view of the problem of crystallization meets their expectations as well. In contrast, the topics for practical projects were usually selected more ‘pragmatically’, *i.e.* students try to grow crystal samples that they can use in their own research.

#### 2.2.4. Resources

The lecture part does not require any special room or resources and can be taught to any number of students. A projector and a computer are sufficient. The lecture part in our case is delivered by several people, each presenting the material which is closer to their research experience. However, all the lectures can be taught by a single lecturer if this is more suitable. If the budget is limited and no laboratory facilities are available, at least this part of the course can be afforded by any university.

The first block of the laboratory exercises does not require any expensive equipment or reagents, but a special room meeting basic safety is still required. Also, someone must take care of the preparation of the experiments and look after the students and their crystallization experiments while they are running. The number of students working simultaneously in the room is limited by the size of the room and the number of tutors. Either the total number of students in this part of the course must be restricted or it is necessary to organize several groups and involve several tutors (or the same tutor will supervise more students in several groups). Still, this part of the course can be also implemented in most universities. Accessories for crystallization using various techniques under ambient conditions (Garcia-Ruiz *et al.*, 2002 ▸) can be useful.

**Figure 2:**
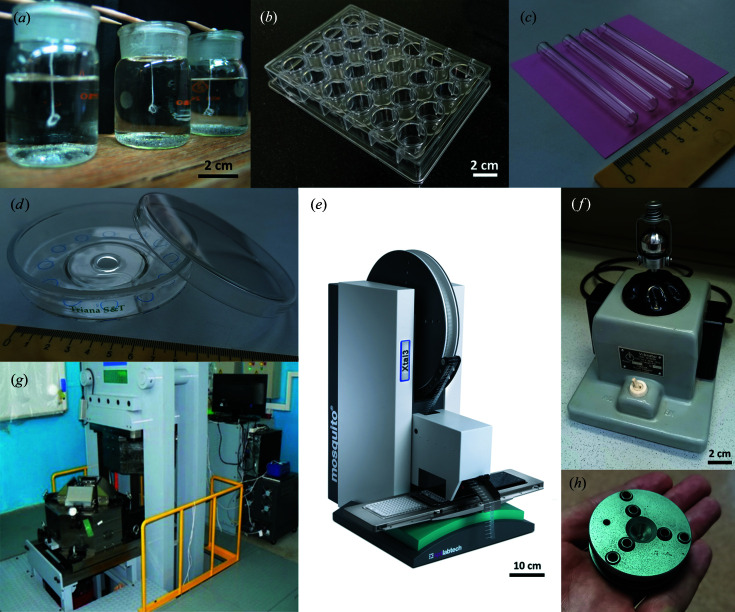
Examples of the experimental setups for the laboratory experiments: (*a*) crystallization from aqueous solutions using seeds, (*b*) 24-well plate (Molecular Dimensions), (*c*) glass tubes for layering diffuse antisolvent crystallization, (*d*) crystallization mushroom (Triana Sci&Tech) (Garcia-Ruíz *et al.*, 2002 ▸), (*e*) Mosquito Xtal3 robot for creating protein crystallization drop set, (*f*) vibrational ball mill (Narva, DDR), (*g*) ‘Discoverer-1500’ DIA-type apparatus at the Sobolev Institute of Geology and Mineralogy SB RAS in Novosibirsk, and (*h*) diamond anvil cell.

The second block of the laboratory exercises, in contrast, requires research instruments and skills that may not be available everywhere. We in Novosibirsk as researchers have access to various instruments and techniques (see Fig. 2 ▸ and Appendix 8). It is therefore not a problem for us to plan and to supervise these individual student research projects. If this is not possible at another institution, the second part of the laboratory exercises can be replaced by a literature analysis in the style of a journal club.

### 2.3. Conclusions and prospects

**Figure 3:**
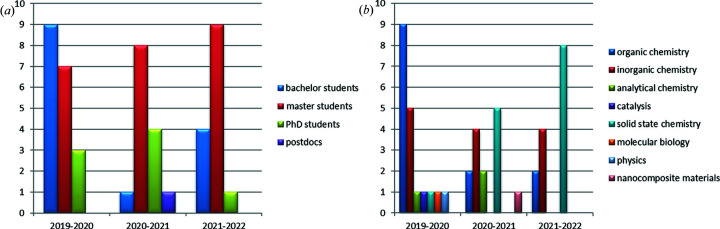
Statistical information on the students who have taken the complete (lectures + practicals) course in the past three years. (*a*) The level of the students and (*b*) the specialities of the students

The first few years of teaching this integrated course have shown that students with very different backgrounds and very different career plans are equally interested in it (Fig. 3 ▸). Whereas the parts of the course related to the basics of crystal growth and to the instrumentation used are rather ‘conservative’ and are similar to what is being taught in many other courses on crystallization, the use of examples selected from earth sciences, life sciences, materials science, chemistry and technology can be varied every year, depending on the interests of the students who have selected the course. We also select new examples from what we can find in recent publications. Those who decide to use our experience can take some of the topics and add others that may be more relevant for their university. We ourselves see the possibilities to modify the course from year to year, including, extending or shortening different blocks depending on the audience and connecting the course with other courses, like solid-state chemistry, X-ray diffraction analysis or biocrystallography. In particular, this course fulfills an important function with the aim of training potential users of mega-science facilities such as synchrotron sources, where crystalline materials and crystals are often required for investigation.

## 3. List of appendices (available as supplementary material)

1. Selected links to the sites of museums and exhibitions related to crystals

2. Selected references to the publications that can be used when preparing the topics listed in Tables 1 ▸–3 ▸ ▸

3. Educational courses given at the Chair of Solid-State Chemistry of the Novosibirsk State University that can be taken by students complementary to the course on Crystal Growth

4. Selected links to the resources related to teaching crystal growth at different levels and for different audiences available on-line

5. Sample plans of selected lectures

a. General introduction: crystals from geology to biology

b. Defects in crystals and crystal growth

c. Melt crystallization and solid-state growth in nature

d. High-pressure crystallization

e. Biocrystallization

6. Descriptions of selected laboratory exercises

a. Crystallization of alums

b. Crystallization of l-ascorbic acid (vitamin C)–l-serine co-crystals by slow evaporation

c. Lysozyme

7. Samples of crystals grown by students

8. List of equipment used when teaching the course

## Supplementary Material

Supporting information file. DOI: 10.1107/S1600576722008032/dv5001sup1.pdf

## Figures and Tables

**Table 1:** The plan of lectures

No.	Topic
1	Introductory lecture *Crystal growth: from mineralogy to biology*.

Basics	
2	Crystal genesis. Basics of nucleation and crystal growth. Crystallization from solution, melt and gas phase. Thermodynamic and kinetic aspects.
3	Equilibrium and non-equilibrium shapes of crystals. Effect of impurities.
4	Defects in crystals and crystal growth.
5	Polymorphism. Isomorphism.
6	Non-classical mechanisms of crystallization.

Crystals and earth sciences	
7	Crystals from lava. Growth of minerals from the silicate melts at the surface and in the depth of the Earth.
8	Granites and basalts. Minerals under pressure. Crystal growth in the deep Earth.
9	Exsolution of solid solutions. Oriented intergrowth: from perthites to ‘stone texts’ (pegmatites).
10	Hydro­thermal crystallization in the Earth’s crust. Opal and agates as interesting examples of silica mineralization.
11	Excursion to the mineralogical museum.

Complex phenomena that can be observed on crystallization	
12	Crystallization at high pressure.
13	Mechanical phenomena accompanying crystallization. Punin–Schtenberg effect.
14	Racemic effects on crystallization. Viedma ripening.
15	Crystallization of small-molecule organic compounds. Pitfalls and challenges.

Crystal growth and life sciences	
16	Crystallization and the pharmaceutical industry.
17	Biomineralization (including pathological biomineralization).
18	Crystallization of biopolymers.

**Table 2:** The topics of the laboratory exercises

No.	Topic
Compulsory for everybody	
Crystallization by slow evaporation of aqueous solutions	
1	Crystallization of potassium and chrome alums by slow evaporation using a seed.
2	Crystallization of sodium chloride from pure aqueous solution and from a solution with urea as impurity. The effect on crystal shape.
3	Crystallization of the polymorphs of glycine. The role of impurities.
4	Crystallization of single crystals of cocrystals. L-Ascorbic acid (vitamin C) with L-serine as a case study.
5	Effect of the crystallization conditions on nucleation and nucleus growth.

Crystallization from gas phase	
6	Crystallization from the gas phase. Benzoic acid as a case study.

Crystallization of poorly soluble compounds	
7	Crystallization of poorly soluble compounds by counter-diffusion. A case study of calcium carbonate.
8	Crystallization of proteins. Hanging-drop crystallization of lysozyme. Crystallization of lysozyme in a capillary.

Optional choices	
9	Crystallization in silica glue. Colored gardens.
10	Viedma ripening (KClO_3_ and γ-glycine as examples).
11	Freeze-drying as a tool of crystallizing highly dispersed but still crystalline particles.
12	Effect of impurities and defects on mechanical properties. Case study of NH_4_Cl (doping with Cu^2+^) and NaCl (whiskers).
13	High-pressure crystallization. Water and benzene as case studies. Recrystallization of β-chlorpropamide into γ-chlorpropamide.
14	Crystallization in gels.
15	Artificial minerals grown at high pressures and temperatures.
16	Lisegang rings and crystallization.
17	Redox crystallization. Copper crystals as a case study.
18	Crystal growth from the melt.
19	Crystallization of proteins using robots.
20	Polymorphism control of pharmaceuticals.
21	Sublimation growth of organic crystals.
22	Obtaining crystalline compounds by mechanochemical techniques. Comparison with crystallization from solution/melt.
23	Crystallization by counter-diffusion versus fast mixing of components. Ni di­methyl­glyoximate, multicomponent crystals in ‘glycine–oxalic acid–water–alcohol’ systems, lead oxalate, calcium carbonate and others as case studies.

**Table 3:** Examples of the titles of student presentations at the final student mini-conferences

No.	Topic
1	Crystals of guanine in living organisms – growth and functions
2	‘Bio-inspired devices’ based on crystals
3	‘Bad’ and ‘good’ impurities in the single crystals of Si – classification, methods of introducing, methods of studying
4	Methods of obtaining quasicrystals
5	Crystallization in gels
6	Thermodynamics and kinetics of the nucleation and growth of the cocrystals of organic compounds
7	Biomineralization
8	Crystals growing in the human body: when are they needed, when are they harmful? How to prevent undesirable crystallization
9	Crystals in chocolate – polymorphism and product quality
10	Impact diamonds – how does their structure reflect the crystallization conditions?
11	Control and fine-tuning the properties of artificial diamonds in the course of their crystallization
12	Crystallization of agate
13	Crystallization in silicate glue – the physical and chemical processes beyond the ‘chemical gardens’
14	Co-crystallization of poorly soluble compounds with MOFs [metal–organic frameworks], in order to find their crystal structures
15	Crystallization of membrane proteins
16	Noble opal as an example of a natural colloid system: structure, optical properties, crystallization
17	Decomposition of solid solutions in minerals
18	How can inclusions in minerals teach us about the history of crystallization?
19	Growth of single crystals during mechanical treatment of polycrystalline and non-crystalline samples
20	Crystallization in confined media
21	Automatic monitoring of the growth of protein crystals
22	Liquid crystals: structures, properties, applications
23	Classical and non-classical mechanisms of crystal growth
24	Hydro­thermal growth of crystals
25	High-pressure crystallization
26	Crystal growth in food industry
27	Crystal growth and pharmaceutical industry
28	Polymorphism control and crystal growth. Disappearing polymorphs
29	Chirality control and crystal growth. Viedma ripening
30	Electrocrystallization
31	High-temperature methods of crystal growth
32	Crystal growth from the gas phase
33	Counterdiffusion and crystallization
34	Technical applications of small and large single crystals
35	Using large-scale facilities to study crystals
36	Crystals used as components of the instrumentation of the large-scale facilities
37	Crystal growth and cements
38	Giant crystals of Naica
