# Supplementary material for: From geology to biology: an interdisciplinary course in crystal growth
Source: J Appl Crystallogr. 2022 Oct 1;55(Pt 5):1368–76. doi: 10.1107/S1600576722008032 (PMC9533743; doi:10.1107/S1600576722008032)
Supplement: Supplementary file 1 [file j-55-01368-sup1.pdf]

JOURNAL OF
APPLIED
CRYSTALLOGRAPHY

Volume 55 (2022)

Supporting information for article:

From geology to biology: an interdisciplinary course in crystal growth

Sergey G. Arkhipov, Tatyana B. Bekker, Anna A. Gaydamaka, Anna Y. Likhacheva, Evgeniy A. Losev and Elena V. Boldyreva

Appendix 1. Selected links to the sites of museums and exhibitions related to crystals (all sites were accessible on the date of submission of the last revision, 29/07/22, at least from Russia, accessibility of some sites may depend on the IP-address of a device)

1. NSU Research and Education Center “Evolution of the Earth” (Novosibirsk, Russia) https://education.nsu.ru/earth_evolution/en/
2. Central Siberian Geological Museum (Novosibirsk, Russia); Центральный Сибирский геологический музей (Новосибирск) https://www.igm.nsc.ru/index.php/ob-institute/struktura/museum
3. A. E. Fersman Mineralogical Museum of Russian Academy of Sciences (Moscow, Russia); Минералогический музей имени А. Е. Ферсмана РАН (Москва) https://fmm.ru/Main_Page?setlang=en
4. Mineralogical Museum of St-Petersburg State University (St-Petersburg, Russia); Минералогический музей Санкт-Петербургского государственного университета http://mmus.geology.spbu.ru/
5. Mining museum (St-Petersburg, Russia) https://museum.spmi.ru/en
6. Mineralogical Museum “Planet” (Ekaterinburg, Russia); Минералогический музей «Планета» (Екатеринбург) https://museum-planeta.ru/
7. Ural Geological Museum of the Ural State Mountain University (Ekaterinburg, Russia); Уральский геологический музей - ФГБОУ ВО "Уральский государственный горный университет" http://ugm.ursmu.ru/
8. Natural Sciences Museum of Ilmen National Park (Miass, Russia); Естественно-научный музей Ильменского заповедника, Миасс https://museum.chelscience.ru/?page_id=3168
9. Museum of Stones (Ekaterinburg, Russia); Екатеринбургский музей камня https://vsemuzei.com/rossiya/muzej-kamnya-i-mineralov-v-ekaterinburge
10. A. V. Sidorov State Mineralogical Museum of the Irkutsk State Research Technical University (Irkutsk, Russia); Государственный Минералогический Музей имени А.В. Сидорова («Иркутский национальный исследовательский технический университет») http://mineral.inrtu.ru/
11. I. V. Belkov Museum of Geology and Mineralogy of the Russian Academy of Sciences (Apatity, Russia); Музей геологии и минералогии им. И.В. Белькова ГИ КНЦ РАН (Апатиты) http://museum.ru/M2304

12. Geological Museum (Novokuzneck, Russia); Геологический музей (Новокузнецк) https://russia.travel/objects/314337/; http://geofondkem.ru/sources/museum-60.pdf?ysclid=l66bmh313p650039147

13. I. K. Bazhenov Mineralogical Museum (Tomsk, Russia); Минералогический музей имени И.К. Баженова (Томск) https://www.tsu.ru/university/museums/minmuseum.php

14. Mineralogical Museum of Tomsk Technical University (Tomsk, Russia); Минералогический музей (Томский политехнический университет) https://tpu.ru/university/meet-tpu/excursion

15. Kolyvan Museum of the History of Stone-Cutting in Altai (Kolyvan, Russia); Колыванский музей истории камнерезного дела на Алтае http://museum.ru/M1497

16. Geological Museum of the Republican National Geological Center (Minsk, Belarus); Геологический музей Республиканского унитарного предприятия «Научно-производственный центр по геологии» (Беларусь) https://geologiya.by/%D0%BC%D1%83%D0%B7%D0%B5%D0%B9/?ysclid=l6697w8v8g801644116

17. Mineralogisches Museum - Philipps-Universität Marburg https://www.uni-marburg.de/en/fb19/minmus

18. Mineralogical Collection Germany at the Krügerhaus (Freiberg) https://www.terra-mineralia.de/english/collections/mineralogical-collection-germany

19. Museum Mineralogia München und Mineralogische Staatssammlung https://www.mineralogische-staatssammlung.de/index.php/en/

20. Museum für Naturkunde Leibniz-Institut für Evolutions- und Biodiversitätsforschung https://www.museumfuernaturkunde.berlin/en

21. Museum für Mineralogie und Geologie (Senckenberg Gesellschaft für Naturforschung) https://www.senckenberg.de/de/institute/senckenberg-naturhistorische-sammlungen-dresden/museum-fuer-mineralogie-und-geologie/

22. Oberharzer Bergwerksmuseum https://www.oberharzerbergwerksmuseum.de/en/

23. Kristalle: Geologisch-Mineralogische Ausstellung der ETH-Zürich http://www.kristalle.ch/sammlung/slidesethz.asp

24. Naturhistorisches Museum Wien https://www.nhm-wien.ac.at/minerale_oesterreichs

25. Cockburn Geological Museum (The University of Edinburgh) https://www.ed.ac.uk/visit/museums-galleries/geology

26. National Museum of Scotland https://www.nms.ac.uk/national-museum-of-scotland/

27. Mineralogy collections (Natural History Museum, London) https://www.nhm.ac.uk/our-science/collections/mineralogy-collections.html
28. Mineral sciences collection (South Australia Museum) https://www.samuseum.sa.gov.au/collection/mineral-sciences
29. Australian Minerals https://www.bukartilla.com.au/australian-minerals-summary
30. Rocks Discovery Museum (Sydney) https://rocksdiscoverymuseum.com/
31. Museo Geominero- Instituto Geológico y Minero de España http://www.igme.es/museo/
32. Musée de Minéralogie des Mines https://www.musee.minesparis.psl.eu/Home/
33. Musée de Bourg d'Oisans http://www.musee-bourgdoisans.fr/
34. Mineral Hall (Royal Belgian Institute of Natural Sciences) https://www.naturalsciences.be/en/museum/exhibitions-view/240/398/395
35. Museu Geológico de Lisboa https://www.mindat.org/museum-338.html
36. Oxford University Museum of Natural History https://oumnh.ox.ac.uk/mineralogy-and-petrology
37. Design museum presents digital crystal exhibition: https://www.montcalm.co.uk/blog/design-museum-presents-digital-crystal-exhibition/
38. “Crystal Dome”: https://kristallwelten.swarovski.com/Content.Node/wattens/Swarovski_Exhibition.en.html

Appendix 2. Selected references to the publications that can be used when preparing the topics listed in Tables 1-3

Basics crystal growth

Belov, N.V. (1977), Real crystal formation, Nauka: Moscow, 233 pp. (in Russian: Белов Н.В. Процессы реального кристаллообразования, Изд. "Наука", М., 1977 г. 233 с.)

Byrappa, K., Keerthiraj, N., & Byrappa, S. M. (2015). Hydrothermal growth of crystals - design and processing. In Handbook of crystal growth (pp. 535-575). Elsevier

Dhanaraj, G., Byrappa, K., Prasad, V., & Dudley, M. (Eds.). (2010). Springer Handbook of Crystal Growth. Springer Science & Business Media. ISBN: 978-3-540-74182-4 e-ISBN: 978-3-540-74761-1, DOI 10.1007/978-3-540-74761-1

Feng, S., & Xu, R. (2001). New materials in hydrothermal synthesis. *Accounts of Chemical Research*, **34**(3), 239-247. https://doi.org/10.1021/ar0000105

Gilman, J. J. (Ed.), The Art and Science of Growing Crystals, Wiley, 1963.

Ivanov, V. K., Fedorov, P. P., Baranchikov, A. Y., Osiko, V. V. (2014). Oriented aggregation of particles: 100 years of investigations of non-classical crystal growth, *Russ. Chem. Rev.*, **83**(12), 1204-1222. DOI: 10.1070/RCR4453; Russian version: В.К. Иванов, П.П. Федоров, А.Е. Баранчиков, В.В. Осико. Ориентированное сращивание частиц: 100 лет исследований неклассического механизма роста кристаллов // Успехи химии. 2014. Т. 83, № 12. С. 1204– 1222

Jones, P. G. (1981). Crystal Growing, *Chemistry in Britain*, **17** (5), 222-225. (https://www.iucr.org/education/teaching-resources/crystal-growing)

Hagiwara, Y., Oaki, Y., & Imai, H. (2021). Wide-area multilayered self-assembly of fluorapatite nanorods vertically oriented on a substrate as a non-classical crystal growth. *Nanoscale*, **13**(21), 9698-9705.

Honigman, B. (1961). Crystal Growth and Form; In Russian: Хонигман Б. Рост и форма кристаллов, ИЛ, 1961. -210 с.

Kalkura, S. N. & Natarajan, S. (2010). Crystallization from gels. In Springer Handbook of Crystal Growth (pp. 1607-1636). Springer, Berlin, Heidelberg

Kozlova, O.G. (1967). Crystal growth, Publishing House of the Moscow State University, 239 pp. (In Russian: Козлова О.Г. Рост кристаллов, М.: Изд-во Моск. ун-та, 1967. - 239 с.)

Kuznetsov, V. D. (1954). Crystals and Crystallization, Gostehizdat, Moscow, 1954 (in Russian: Кузнецов, В. Д. Кристаллы и кристаллизация, Гостехиздат, 1954)

Laudise, R. A. (2004). Hydrothermal synthesis of crystals. *50 years Progress in Crystal Growth Reprint Collection*, Feigelson, R.S. (Ed.), p. 185. ISBN: 0 444 51650-6

Laudise, R. A., & Parker, R. L. (1970). Crystal Growth Mechanisms, Academic Press, New York, 540 pp.; In Russian: Лодиз Р., Паркер Р. Рост монокристаллов, Москва, Мир, 1974, 540 с.

Markov, I.V. (2016). Crystal Growth for Beginners. World Scientific, 2016-06-05. — ISBN 978-981-314-342-5, 978-981-314-385-2.

Mergo III, J. C. & Seto, J. (2020). On Simulating the Formation of Structured, Crystalline Systems via Non-classical Pathways. *Frontiers in Materials*, **7**, 75. https://doi.org/10.3389/fmats.2020.00075

Pamplin, B. R. (Ed.). (2013). Crystal Growth: International Series on the Science of the Solid State. Elsevier.

Petrov, T.G., Treivus, E.B., Punin, Yu.O., Kasatkin, A.P. (1983). Crystal Growth from Solutions, Nedra: Leningrad, 200 pp.; in Russian: Т. Г. Петров, Е. Б. Трейвус, Ю. О. Пунин, А. П. Касаткин (1983). Выращивание кристаллов из растворов, Недра, Ленинград, 200 стр.

Portnov, V. N. (2013). The Effect of Impurities on the Rate of Growth of Crystal Faces on Crystallization from Solution, Publishing House of the Lobachevskii University of Nizhnii Novgorod; In Russian: Портнов В.Н., Влияние примесей на скорость роста граней кристаллов из раствора: Нижний Новгород: Изд-во Нижегородского госуниверситета им. Н.И. Лобачевского, 2013, 166 с.

Physics of Crystal Growth. — Cambridge: Cambridge University Press, 1999. — 1 online resource (400 pages) c. — ISBN 978-0-511-62252-6, 0-511-62252-X, 978-0-521-55198-4, 0-521-55198-6.

Punin Yu. O., Shtukenberg, A.G. (2007), Autodeformation Defects in Crystals, Publishing House of the S-Petersbourg State University, 316 pp; In Russian: Ю.О. Пунин, А.Г. Штукенберг, Автодеформационные дефекты кристаллjd. Изд-во СПбГУ, СПб, 2007, 316 стр., ISBN 978-5-288-04759-6

Robert, M. C., Vidal, O., Garcia-Ruiz, J. M., & Otalora, F. (1999). Crystallization in Gels and Related. In: Crystallization of Nucleic Acids and Proteins: A Practical Approach, Ed. by Ducruix, A. & Giege, R, pp. 149-177; ISBN 0-19-963678-8. http://www.oup.co.uk/PAS

Shaskolskaia, M. P. (1984), Crystallography, Vysshaya Shkola: Moscow, 376 pp.; In Russian: Шаскольская М.П. Кристаллография, Учеб. пособие для втузов. 2-е изд., перераб. и доп. - М.: Высшая шк. , 1984. - 376 с.

Sheftal' N.N. (1976) Trends in Real Crystal Formation and Some Principles for Single Crystal Growth. In: Sheftal' N.N. (eds) Growth of Crystals. Springer, Boston, MA. https://doi.org/10.1007/978-1-4613-4256-4_17

Shtukenberg, A. G., García-Ruiz, J. M., & Kahr, B. (2021). Punin Ripening and the Classification of Solution-Mediated Recrystallization Mechanisms. *Crystal Growth & Design*, **21**(2), 1267-1277. https://doi.org/10.1021/acs.cgd.0c01545

Shubnikov, A. V. (1935). How do Crystals Grow? Publishing House of the USSR Academy of Sciences, 176 pp.; In Russian: Шубников А.В. Как растут кристаллы, АН СССР. М. -Л. 1935. 176 с.

Shubnikov, A. V. (1947). Crystal Growth, Publishing House of the USSR Academy of Sciences, 74 pp.; In Russian: Шубников А.В. Образование кристаллов, М.-Л.: Изд. АН СССР, 1947. - 74 с.

Spiridonov E.M. (2019). Phenomena of crystal splitting during growth as a result of the Sternberg-Punin and Rebinder effects, *Doklady Earth Sciences*, **485**(2), 426–427; In Russian: Доклады Академии наук. - 2019. - Vol. 485. - N. 5. - P. 619-620. doi: 10.31857/S0869-56524855619-620. doi: 10.31857/S0869-56524855619-620

Timofeeva, V. A. (1978). Crystal Growth from Solutions and Melts, Nauka: Moscow, 268 pp; In Russian: Тимофеева В.А. Рост кристаллов из растворов и расплавов, М.: Наука, 1978. -268 с.

Wilke, K. Th. (1963). Methoden der Kristallzüchtung, VEB Deutscher Verlag der Wissenschaften, Berlin; In Russian: Вильке К.-Т. Выращивание кристаллов, Л. Недра, 1977, 602 стр.

Wood, E.A. (1972), Crystals - a Handbook for School Teachers (https://www.iucr.org/education/pamphlets/20)

De Yoreo, J. J., Gilbert P. U. P. A., Sommerdijk, N. A. J. M., Lee Penn, R., Whitelam, S., Joester, D., Zhang, H., Rimer, J. D., Navrotsky, A., Banfield, J. F., Wallace, A. F., Marc Michel F., Meldrum, F. C., Cölfen, H., Dove, P. M. (2015). Crystallization by particle attachment in synthetic, biogenic, and geologic environments, *Science*, **349**, N 6247. P. aaa6760. DOI: 10.1126/science.aaa6760

Crystal growth and earth sciences

Akaogi, M., Navrotsky, A., Yagi, T., & Akimoto, S. I. (1987). Pyroxene-garnet transformation: Thermochemistry and elasticity of garnet solid solutions, and application to a

pyrolite mantle. *High-Pressure Research in Mineral Physics, Geophys. Monogr. Se*r, **39**, 251-260. ISBN 0-87590-066-6

Asimow, P. D., Lin, C., Bindi, L., Ma, C., Tschauner, O., Hollister, L. S., & Steinhardt, P. J. (2016). Shock synthesis of quasicrystals with implications for their origin in asteroid collisions. *Proceedings of the National Academy of Sciences*, **113**(26), 7077-7081. https://doi.org/10.1073/pnas.1600321113

Bindi, L., Steinhardt, P. J., Yao, N., & Lu, P. J. (2009). Natural quasicrystals, *Science*, **324**(5932), 1306-1309. DOI: 10.1126/science.1170827

Bonazzi, M., Tumiati, S., Thomas, J. B., Angel, R. J., & Alvaro, M. (2019). Assessment of the reliability of elastic geobarometry with quartz inclusions. *Lithos*, **350**, 105201. https://doi.org/10.1016/j.lithos.2019.105201

Cesare, B., Parisatto, M., Mancini, L., Peruzzo, L., Franceschi, M., Tacchetto, T., Reddy, S., Spiess, R., Nestola, F. & Marone, F. (2021). Mineral inclusions are not immutable: Evidence of post-entrapment thermally-induced shape change of quartz in garnet. *Earth and Planetary Science Letters*, **555**, 116708. https://doi.org/10.1016/j.epsl.2020.116708

Deniskina, M. D., Kalinin, D. V., Jazantseva, L. K. (1980). Noble opals, their synthesis and genesis in Nature, Nauka, Novosibirsk, 64 pp; In Russian: Н.Д. Денискина, Д.В. Калинин, Л.К. Казанцева. Благородные опалы, их синтез и генезис в природе. Наука: Новосибирск, 1980, 64 стр. https://www.geokniga.org/bookfiles/geokniga-blagorodnye-opaly-ih-sintez-i-genezis-v-prirode.pdf

Van Driessche, A. E., García-Ruíz, J. M., Tsukamoto, K., Patino-Lopez, L. D., & Satoh, H. (2011). Ultraslow growth rates of giant gypsum crystals. *Proceedings of the National Academy of Sciences*, **108**(38), 15721-15726. https://doi.org/10.1073/pnas.1105233108

Ferrero, S., & Angel, R. J. (2018). Micropetrology: Are inclusions grains of truth? *Journal of Petrology*, **59**(9), 1671-1700. https://doi.org/10.1093/petrology/egy075

García-Ruiz, J. M., Villasuso, R., Ayora, C., Canals, A., & Otálora, F. (2007). Formation of natural gypsum megacrystals in Naica, Mexico. *Geology*, **35**(4), 327-330. https://doi.org/10.1130/G23393A.1

Charles A. Geiger (2016) A tale of two garnets: The role of solid solution in the development toward a modern mineralogy, *American Mineralogist: Journal of Earth and Planetary Materials*, **101**, 1735–1749. https://doi.org/10.2138/am-2016-5522

Gilio, M., Angel, R. J., & Alvaro, M. (2021). Elastic geobarometry: How to work with residual inclusion strains and pressures. *American Mineralogist: Journal of Earth and Planetary Materials*, **106**(9), 1530-1533. https://doi.org/10.2138/am-2021-7928

Godovikov, A. A., Ripinen, O. I., Motorin, S. G. (1987). Agates. Nedra: Moscow, 368 pp,; In Russian: Годовиков А.А., Рипинен О.И., Моторин С.Г. Агаты. М.: Недра, 1987. - 368 с.

Götze, J., Möckel, R., & Pan, Y. (2020). Mineralogy, geochemistry and genesis of agate—A review. *Minerals*, **10**(11), 1037. https://doi.org/10.3390/min10111037

Götze, J., Plötze, M., Tichomirowa, M., Fuchs, H., & Pilot, J. (2001). Aluminium in quartz as an indicator of the temperature of formation of agate. *Mineralogical Magazine*, **65**(3), 407-413. https://doi.org/10.1180/002646101300119484

Grigoriev, D. P., Zhabin, A. G. (1979) Ontogenia of minerals; In Russian: Григорьев Д.П., Жабин А.Г. "Онтогения минералов", Наука, Москва, 275 стр.; https://geo.web.ru/; https://www.geokniga.org/labels/28725

Heaney, P. J., & Davis, A. M. (1995). Observation and origin of self-organized textures in agates. *Science*, **269**(5230), 1562-1565. DOI: 10.1126/science.269.5230.1562

M.B. Holness (2014). The effect of crystallization time on plagioclase grain shape in dolerites, *Contrib Mineral Petrol,* **168**, 1076. DOI 10.1007/s00410-014-1076-5

Howell, D., Griffin, W. L., Yang, J., Gain, S., Stern, R. A., Huang, J. X., Jacob, D.E., Xud, X., Stokes, A. J., O'Reilly, S. Y. & Pearson, N. J. (2015). Diamonds in ophiolites: Contamination or a new diamond growth environment? *Earth and Planetary Science Letters*, **430**, 284-295. https://doi.org/10.1016/j.epsl.2015.08.023

Ito, E., & Takahashi, E. (1987). Ultrahigh-pressure phase transformations and the constitution of the deep mantle. In High-pressure research in mineral physics (Vol. 39, pp. 221-229). American Geophysical Union Washington, DC, ISBN 0-87590-066-6

Kantor B. Z. (2003). Crystal Growth and Development Interpreted from a Mineral's Present Form, *Mineralogical Almanac*. Vol. 6. http://mindraw.web.ru/Kant-StAg.htm

Kigai, I. N. (2017). Conditions of the agates formation, *Mineralogy*, **2**, 75-90; In Russian: И.Н. Кигай (2017) Об условиях образования агатов, Минералогия, №2, 75-90.

Koeberl, C., Masaitis, V. L., Shafranovsky, G. I., Gilmour, I., Langenhorst, F., & Schrauder, M. (1997). Diamonds from the Popigai impact structure, Russia. *Geology*, **25**(11), 967-970

Masaitis, V. L., Shafranovskii, G. I., Ezerskii, V. A., & Reshetniak, N. B. (1990). Impact diamonds in ureilites and impactites. *Meteoritika*, **49**, 180-196 (ISSN 0369-2507), in Russian

Mason, R., Burton, K.W., Yuan, Y., She, Z. (2010). Chiastolite. *Gondwana Research*, **18**, 222-229. https://doi.org/10.1016/j.gr.2010.03.005

Mazzucchelli, M. L., Angel, R. J., & Alvaro, M. (2021). EntraPT: An online platform for elastic geothermobarometry, *American Mineralogist: Journal of Earth and Planetary Materials*, 106(5), 830-837. https://doi.org/10.2138/am-2021-7693CCBYNCND

Moxon, T., & Palyanova, G. (2020). Agate genesis: A continuing enigma. *Minerals*, **10**(11), 953. https://doi.org/10.3390/min10110953

Nestola, F., Goodrich, C. A., Morana, M., Barbaro, A., Jakubek, R. S., Christ, O., Brenker, F.E., M. Chiara Domeneghetti, Dalconi, M. C., Alvaro, M., Fioretti, A. M., Litasov, K. D., Fries, M. D., Leoni, M., Casati, N. P. M., Jenniskens, P. & Shaddad, M. H. (2020). Impact shock origin of diamonds in ureilite meteorites. *Proceedings of the National Academy of Sciences*, **117**(41), 25310-25318. https://doi.org/10.1073/pnas.1919067117

Ohfuji, H., Irifune, T., Litasov, K.D., Yamashita, T., Isobe, F., Afanasiev, V.P. & Pokhilenko, N.P. (2015). Natural occurrence of pure nano-polycrystalline diamond from impact crater. *Sci. Rep*. **5**, 14702. https://doi.org/10.1038/srep14702

Otálora, F., & García-Ruiz, J. (2014). Nucleation and growth of the Naica giant gypsum crystals. *Chem. Soc. Rev.* **43**(7), 2013-2026. https://doi.org/10.1039/C3CS60320B

Palyanov, Y. N., Borzdov, Y. M., Kupriyanov, I. N., Khokhryakov, A. F., & Nechaev, D. V. (2015). Diamond crystallization from an Mg–C system under high pressure, high temperature conditions. *CrystEngComm*, **17**(26), 4928-4936. https://doi.org/10.1039/C5CE00897B

Palyanov, Y. N., Borzdov, Y. M., Sokol, A. G., Bataleva, Y. V., Kupriyanov, I. N., Reutsky, V. N., Wiedenbeck, M. & Sobolev, N. V. (2021). Diamond formation in an electric field under deep Earth conditions. *Science Advances*, **7**(4), eabb4644. DOI: 0.1126/sciadv.abb4644

Palyanov, Y. N., Kupriyanov, I. N., Borzdov, Y. M., & Bataleva, Y. V. (2015). High-pressure synthesis and characterization of diamond from an Mg–Si–C system. *CrystEngComm*, **17**(38), 7323-7331. https://doi.org/10.1039/C5CE01265A

Palyanov, Y. N., Kupriyanov, I. N., Khokhryakov, A. F., & Ralchenko, V. G. (2015). Crystal growth of diamond. In *Handbook of Crystal Growth* (pp. 671-713). Elsevier

Palyanova, G. (2021). Editorial for Special Issue "Agates: Types, Mineralogy, Deposits, Host Rocks, Ages and Genesis". *Minerals*, **11**(10), 1035. https://doi.org/10.3390/min11101035

Patnis, A., & McConnell, J. D. C. (1980). Principles of mineral behavior; In Russian: A. Патнис, Дж. Мак-Коннел. Основные черты поведения минералов. Москва: Мир, 1983. - 304 с.

Popov V. S. (1995). Earth magmatism, *Soros Educational Journal*, **1**, 74-81; In Russian: B.C. Попов (1995) Магматизм Земли. Соросовский Образовательный Журнал, №1,74-81. http://popovgeo.sfedu.ru/

Popov, V. A. & Popova, V. I. (2006). Mineralogy of the pegmatites of the Ilmen mountains, 59 pp. In Russian: Попов В.А., Попова В.И., Минералогия пегматитов Ильменских гор, 2006. 59 стр.

Putnis, A., McConnell, J. D. C. (1980). Principles of mineral behavior. Geoscience texts; v. 1, New York, Elsevier, 257 pp.

Ringwood, A. E. (1991). Phase transformations and their bearing on the constitution and dynamics of the mantle. *Geochimica et Cosmochimica Acta*, **55**(8), 2083-2110. https://doi.org/10.1016/0016-7037(91)90090-R

Sveshnikova, O.L. (2016). Minerals containing quartz, Exhibition in the Fersman Mineralogical Museum of Russian Academy of Sciences, *New data about minerals*, Issue 51, pp. 1340145; In Russian: О.Л. Свешникова (2016) Выставка «Минералы хрусталеносных кварцевых жил» в минералогическом музее им. А.Е. Ферсмана Российской Академии Наук // Новые данные о минералах. Вып. 51, с. 134-145.

Shumilova, T. G., Isaenko, S. I., Ulyashev, V. V., Kazakov, V. A., & Makeev, B. A. (2018). After-coal diamonds: an enigmatic type of impact diamonds. *European Journal of Mineralogy*, **30**(1), 61-76. https://doi.org/10.1127/ejm/2018/0030-2715

M.-L.C. Sirbescu, C. Schmidt, I.V. Veksler, A.G. Whittington, M. Wilke (2017) Experimental Crystallization of Undercooled Felsic Liquids: Generation of Pegmatitic Texture. *Journal of Petrology*, **58**(3), 539–568. doi: 10.1093/petrology/egx027

Sonin, V., Zhimulev, E., Chepurov, A., Gryaznov, I., Chepurov, A., Afanasiev, V., & Pokhilenko, N. (2021). Experimental Etching of Diamonds: Extrapolation to Impact Diamonds from the Popigai Crater (Russia). *Minerals*, **11**(11), 1229. https://doi.org/10.3390/min11111229

Tommasini, S., Bindi, L., Petrelli, M., Asimow, P. D., & Steinhardt, P. J. (2021). Trace Element Conundrum of Natural Quasicrystals. *ACS Earth and Space Chemistry*, **5**(3), 676-689. https://doi.org/10.1021/acsearthspacechem.1c00004

Wang, Y., & Merino, E. (1990). Self-organizational origin of agates: Banding, fiber twisting, composition, and dynamic crystallization model. *Geochimica et Cosmochimica Acta*, **54**(6), 1627-1638. https://doi.org/10.1016/0016-7037(90)90396-3

De Yoreo, J. J., Gilbert P. U. P. A., Sommerdijk, N. A. J. M., Lee Penn, R., Whitelam, S., Joester, D., Zhang, H., Rimer, J. D., Navrotsky, A., Banfield, J. F., Wallace, A. F., Marc Michel F., Meldrum, F. C., Cölfen, H., Dove, P. M. (2015). Crystallization by particle attachment in synthetic, biogenic, and geologic environments, *Science*, **349**, N 6247. P. aaa6760. DOI: 10.1126/science.aaa6760

Crystal growth and materials

Ashfold, M. N., Goss, J. P., Green, B. L., May, P. W., Newton, M. E., & Peaker, C. V. (2020). Nitrogen in diamond. *Chem. Rev.*, **120**(12), 5745-5794. https://doi.org/10.1021/acs.chemrev.9b00518

Cai, Z., Li, Z., Ravaine, S., He, M., Song, Y., Yin, Y., Zheng, H., Teng, J. & Zhang, A. (2021). From colloidal particles to photonic crystals: advances in self-assembly and their emerging applications. *Chem. Soc. Rev.,* **50**, 5898-5951. https://doi.org/10.1039/D0CS00706D

Graff, K. (2013). *Metal impurities in silicon-device fabrication* (Vol. 24). Springer Science & Business Media, 270 pp. ISBN 978-3-642-62965-5

Han, J. H., Shneidman, A. V., Kim, D. Y., Nicolas, N. J., van der Hoeven, J. E., Aizenberg, M., & Aizenberg, J. (2022). Highly Ordered Inverse Opal Structures Synthesized from Shape-Controlled Nanocrystal Building Blocks. **61**(3), e202111048. https://doi.org/10.1002/anie.202111048

Ivanov, E. Y., Konstanchuk, I. G., Bokhonov, B. D., & Boldyrev, V. V. (1989). Mechanochemical synthesis of icosahedral phases in Mg-Zn-Al and Mg-Cu-Al alloys. *Reactivity of Solids*, **7**(2), 167-172. https://doi.org/10.1016/0168-7336(89)80026-9

Keys, A. S., & Glotzer, S. C. (2007). How do quasicrystals grow? *Physical Review Letters*, **99**(23), 235503. https://doi.org/10.1103/PhysRevLett.99.235503

Kissinger, G., & Pizzini, S. (Eds.). (2014). Silicon, germanium, and their alloys: growth, defects, impurities, and nanocrystals. CRC Press, 431 pp. ISBN-13:978-1-1665-8664-2. https://doi.org/10.1201/b17868

Konstanchuk, I. G., Ivanov, E. Y., Bokhonov, B. B., & Boldyrev, V. V. (2001). Hydriding properties of mechanically alloyed icosahedral phase $Ti_{45}Zr_{38}Ni_{17}$. *Journal of Alloys and Compounds*, **319**(1-2), 290-295. https://doi.org/10.1016/S0925-8388(01)00911-2

Napolskii, K. S., Sapoletova, N. A., Gorozhankin, D. F., Eliseev, A. A., Chernyshov, D. Y., Byelov, D. V., Grigoryeva, N. A., Mistonov, A. A., Bouwman, W. G., Kvashnina, K. O., Lukashin, A. V., Snigirev, A.A., Vassilieva, A. V., Grigoriev, S. V. & Petukhov, A. V. (2010). Fabrication of artificial opals by electric-field-assisted vertical deposition. *Langmuir*, **26**(4), 2346-2351. https://doi.org/10.1021/la902793b

Palyanov, Y. N., Kupriyanov, I. N., Borzdov, Y. M., & Surovtsev, N. V. (2015). Germanium: a new catalyst for diamond synthesis and a new optically active impurity in diamond. *Scientific Reports*, **5**(1), 1-8. https://doi.org/10.1038/srep14789

Ruhl, T., Spahn, P., & Hellmann, G. P. (2003). Artificial opals prepared by melt compression. *Polymer*, **44**(25), 7625-7634. https://doi.org/10.1016/j.polymer.2003.09.047

Shevyrtalov, S., Barannikov, A., Palyanov, Y., Khokhryakov, A., Borzdov, Y., Sergueev, I., Rashchenko, S. V. & Snigirev, A. (2021). Towards high-quality nitrogen-doped diamond single crystals for X-ray optics. *Journal of Synchrotron Radiation*, 28(1), 104-110. https://doi.org/10.1107/S1600577520014538

Voronkov, V. V., & Falster, R. (2002). Intrinsic point defects and impurities in silicon crystal growth. *Journal of the Electrochemical Society*, **149**(3), G167. https://doi.org/10.1149/1.1435361

Yoshida, Y., & Langouche, G. (Eds.) (2015). Defects and Impurities in Silicon Materials. Berlin: Springer. ISBN: 978-4-431-55800-2

Crystals of proteins and other biopolymers

Garcıa-Ruiz, J. M. (2003). Nucleation of protein crystals. *Journal of Structural Biology*, **142**(1), 22-31. https://doi.org/10.1016/S1047-8477(03)00035-2

Garcıa-Ruiz, J. M., Gavira, J. A., Otálora, F., Guasch, A., & Coll, M. (1998). Reinforced protein crystals. *Materials Research Bulletin*, **33**(11), 1593-1598. https://doi.org/10.1016/S0025-5408(98)00172-X

Gavira, J. A., & García-Ruiz, J. M. (2002). Agarose as crystallisation media for proteins II: trapping of gel fibres into the crystals. *Acta Crystallographica Section D: Biological Crystallography*, **58**(10), 1653-1656. https://doi.org/10.1107/S0907444902014609

Guo, Y. (2020). Be cautious with crystal structures of membrane proteins or complexes prepared in detergents. *Crystals*, **10**(2), 86. https://doi.org/10.3390/cryst10020086

Kato, R., Hiraki, M., Yamada, Y., Tanabe, M., & Senda, T. (2021). A fully automated crystallization apparatus for small protein quantities. *Acta Crystallographica Section F: Structural Biology Communications*, **77**(1), 29-36. https://doi.org/10.1107/S2053230X20015514

Kermani, A. A. (2021). A guide to membrane protein X-ray crystallography. *The FEBS journal*, **288**(20), 5788-5804. https://doi.org/10.1111/febs.15676

Kühlbrandt, W. (1988). Three-dimensional crystallization of membrane proteins. *Quarterly Reviews of Biophysics*, **21**(4), 429-477. https://doi.org/10.1017/S0033583500004625

Landau, E. M., & Rosenbusch, J. P. (1996). Lipidic cubic phases: a novel concept for the crystallization of membrane proteins. *Proceedings of the National Academy of Sciences*, **93**(25), 14532-14535. https://doi.org/10.1073/pnas.93.25.14532

Newby, Z. E., D O'Connell, J., Gruswitz, F., Hays, F. A., Harries, W. E., Harwood, I. M., Ho, J. D., Lee, J. K., Savage, D. F., Miercke, L. J. W. & Stroud, R. M. (2009). A general protocol for the crystallization of membrane proteins for X-ray structural investigation. *Nature Protocols*, **4**(5), 619-637. https://doi.org/10.1038/nprot.2009.27

Ostermeier, C., & Michel, H. (1997). Crystallization of membrane proteins. *Current Opinion in Structural Biology*, **7**(5), 697-701. https://doi.org/10.1016/S0959-440X(97)80080-2

Otálora, F., Gavira, J. A., Ng, J. D., & García-Ruiz, J. M. (2009). Counterdiffusion methods applied to protein crystallization. *Progress in Biophysics and Molecular Biology*, **101**(1-3), 26-37. https://doi.org/10.1016/j.pbiomolbio.2009.12.004

Ren, Z., Wang, C., Shin, H., Bandara, S., Kumarapperuma, I., Ren, M. Y., Kang, W. & Yang, X. (2020). An automated platform for *in situ* serial crystallography at room temperature. *IUCrJ*, **7**(6), 1009-1018. https://doi.org/10.1107/S2052252520011288

Sauter, C., Otálora, F., Gavira, J. A., Vidal, O., Giegé, R., & García-Ruiz, J. M. (2001). Structure of tetragonal hen egg-white lysozyme at 0.94 Å from crystals grown by the counter-diffusion method. *Acta Crystallographica Section D: Biological Crystallography*, **57**(8), 1119-1126. https://doi.org/10.1107/S0907444901008873

Stiers, K. M., Lee, C. B., Nix, J. C., Tanner, J. J., & Beamer, L. J. (2016). Synchrotron-based macromolecular crystallography module for an undergraduate biochemistry laboratory course. *Journal of Applied Crystallography*, **49**(6), 2235-2243. https://doi.org/10.1107/S1600576716016800

Subedi, S., Dinc, I., Tran, T. X., Sharma, D., Shrestha, B. R., Pusey, M. L., & Aygun, R. S. (2020). Visual-x2: interactive visualization and analysis tool for protein crystallization. *Network Modeling Analysis in Health Informatics and Bioinformatics*, **9**(1), 1-13. https://doi.org/10.1007/s13721-020-0220-6

Bioinspired crystals. Biomineralization. Crystallization and origin of life

Aizenberg, J. (2004). Crystallization in patterns: a bio-inspired approach. *Advanced Materials*, **16**(15), 1295-1302. https://doi.org/10.1002/adma.200400759

Bizzarri, B. M., Saladino, R., Delfino, I., García-Ruiz, J. M., & Di Mauro, E. (2021). Prebiotic organic chemistry of formamide and the origin of life in planetary conditions: What

we know and what is the future. *International Journal of Molecular Sciences*, **22**(2), 917. https://doi.org/10.3390/ijms22020917

Bogdanov, J. Yu. (1986). A Physicist as a Guest of a Biologists, Nauka: Moscow, 140 pp.; In Russian: Богданов К.Ю. Физик в гостях у биолога, Москва, Наука, 1986, 140 с.

Boskey, A. L. (1998). Biomineralization: conflicts, challenges, and opportunities. *Journal of Cellular Biochemistry*, **72**(S30–31), 83-91. https://doi.org/10.1002/(SICI)1097-4644(1998)72:30/31+<83::AID-JCB12>3.0.CO;2-F

Chen, X., Luo, L., Zeng, Z., Jiao, J., Shehzad, M., Yuan, G., Luo, H. & Wang, Y. (2020). Bio-inspired flexible vibration visualization sensor based on piezo-electrochromic effect. *Journal of Materiomics*, **6**(4), 643-650. https://doi.org/10.1016/j.jmat.2020.06.002

Ehrlich, H., Bailey, E., Wysokowski, M., & Jesionowski, T. (2021). Forced biomineralization: a review. *Biomimetics*, **6**(3), 46. https://doi.org/10.3390/biomimetics6030046

Fu, Y., Tippets, C. A., Donev, E. U., & Lopez, R. (2016). Structural colors: from natural to artificial systems. *Wiley Interdisciplinary Reviews: Nanomedicine and Nanobiotechnology*, **8**(5), 758-775. https://doi.org/10.1002/wnan.1396

García Ruiz, J. M., Carnerup, A., Christy, A. G., Welham, N. J., & Hyde, S. T. (2002). Morphology: an ambiguous indicator of biogenicity. *Astrobiology*, **2**(3), 353-369. https://doi.org/10.1089/153110702762027925

García-Ruiz, J. M., Nakouzi, E., Kotopoulou, E., Tamborrino, L., & Steinbock, O. (2017). Biomimetic mineral self-organization from silica-rich spring waters. *Science Advances*, **3**(3), e1602285. DOI: 10.1126/sciadv.1602285

Gur, D., Palmer, B. A., Weiner, S., & Addadi, L. (2017). Light manipulation by guanine crystals in organisms: biogenic scatterers, mirrors, multilayer reflectors and photonic crystals. *Advanced Functional Materials*, **27**(6), 1603514. https://doi.org/10.1002/adfm.201603514

Gur, D., Pierantoni, M., Elool Dov, N., Hirsh, A., Feldman, Y., Weiner, S., & Addadi, L. (2016). Guanine crystallization in aqueous solutions enables control over crystal size and polymorphism. *Crystal Growth & Design*, **16**(9), 4975-4980. https://doi.org/10.1021/acs.cgd.6b00566

Hirsch, A., Gur, D., Polishchuk, I., Levy, D., Pokroy, B., Cruz-Cabeza, A. J., Addadi, L., Kronik, L. & Leiserowitz, L. (2015). "Guanigma": The revised structure of biogenic anhydrous guanine. *Chemistry of Materials*, **27**(24), 8289-8297. https://doi.org/10.1021/acs.chemmater.5b03549

Jantschke, A., Pinkas, I., Hirsch, A., Elad, N., Schertel, A., Addadi, L., & Weiner, S. (2019). Anhydrous β-guanine crystals in a marine dinoflagellate: Structure and suggested function. *Journal of Structural Biology*, **207**(1), 12-20. https://doi.org/10.1016/j.jsb.2019.04.009

Kapishnikov, S., Hempelmann, E., Elbaum, M., Als-Nielsen, J., & Leiserowitz, L. (2021). Malaria Pigment Crystals: The Achilleso Heel of the Malaria Parasite. *ChemMedChem*, **16**, 1515-1532. doi.org/10.1002/cmdc.202000895

Kimura, T., Takasaki, M., Hatai, R., Nagai, Y., Uematsu, K., Oaki, Y., Osada, M., Tsuda, H., Ishigure, T., Toyofuku, T., Shimode, S. & Imai, H. (2020). Guanine crystals regulated by chitin-based honeycomb frameworks for tunable structural colors of sapphirinid copepod, Sapphirina nigromaculata. *Scientific Reports*, **10**(1), 1-7. https://doi.org/10.1038/s41598-020-59090-4

Konigsberger, E., & Konigsberger, L. (Eds.). (2006). Biomineralization: medical aspects of solubility. John Wiley & Sons, 302 pp. ISBN-10:0-470-09209-2

Kuang, M., Wang, J., & Jiang, L. (2016). Bio-inspired photonic crystals with superwettability. *Chemical Society Reviews*, **45**(24), 6833-6854. https://doi.org/10.1039/C6CS00562D

Levy-Lior, A., Pokroy, B., Levavi-Sivan, B., Leiserowitz, L., Weiner, S., & Addadi, L. (2008). Biogenic guanine crystals from the skin of fish may be designed to enhance light reflectance. *Crystal Growth and Design*, **8**(2), 507-511. https://doi.org/10.1021/cg0704753

Mann, S. (1988). Molecular recognition in biomineralization. *Nature*, **332**(6160), 119-124. https://doi.org/10.1038/332119a0

Nudelman, F., & Sommerdijk, N. A. (2012). Biomineralization as an inspiration for materials chemistry. *Angewandte Chemie International Edition*, **51**(27), 6582-6596. https://doi.org/10.1002/anie.201106715

Oaki, Y., Kaneko, S., & Imai, H. (2012). Morphology and orientation control of guanine crystals: a biogenic architecture and its structure mimetics. *Journal of Materials Chemistry*, **22**(42), 22686-22691. https://doi.org/10.1039/C2JM33047D

Sivaguru, M., Saw, J. J., Williams, J. C., Lieske, J. C., Krambeck, A. E., Romero, M. F., Chia, N., Schwaderer, A. L., Alcalde, R. E., Bruce, W. J., Wildman, D. E., Fried, G. A., Werth, C. J., Reeder, R. J., Yau, P. M., Sanford, R. A. & Fouke, B. W. (2018). Geobiology reveals how human kidney stones dissolve *in vivo*. *Scientific Reports*, **8**(1), 1-9. https://doi.org/10.1038/s41598-018-31890-9

Sivaguru, M., Saw, J. J., Wilson, E. M., Lieske, J. C., Krambeck, A. E., Williams, J. C., Romero, M. F., Fouke, K. W., Curtis, M. W., Kear-Scott, J. L., Chia, N. & Fouke, B. W.

(2021). Human kidney stones: a natural record of universal biomineralization. *Nature Reviews Urology*, 1-29. https://doi.org/10.1038/s41585-021-00469-x

Solomonov, I., Osipova, M., Feldman, Y., Baehtz, C., Kjaer, K., Robinson, I. K., Webster, G. T., McNaughton, D., Wood, B. R., Weissbuch, I. & Leiserowitz, L. (2007). Crystal nucleation, growth, and morphology of the synthetic malaria pigment β-hematin and the effect thereon by quinoline additives: the malaria pigment as a target of various antimalarial drugs. *Journal of the American Chemical Society*, **129**(9), 2615-2627. https://doi.org/10.1021/ja0674183

Su, H., Song, F., Dong, Q., Li, T., Zhang, X., & Zhang, D. (2011). Bio-inspired synthesis of ZnO polyhedral single crystals under eggshell membrane direction. *Applied Physics A*, **104**(1), 269-274. https://doi.org/10.1007/s00339-010-6122-1

Weiner, S. (2008). Biomineralization: a structural perspective. *Journal of Structural Biology*, **163**(3), 229-234. https://doi.org/10.1016/j.jsb.2008.02.001

Weiner, S., & Addadi, L. (2011). Crystallization pathways in biomineralization. *Annual Review of Materials Research*, **41**, 21-40. https://doi.org/10.1146/annurev-matsci-062910-095803

Wendt, C., de Souza, W., Pinheiro, A., Silva, L., de Sá Pinheiro, A. A., Gauvin, R., & Miranda, K. (2021). High-Resolution Electron Microscopy Analysis of Malaria Hemozoin Crystals Reveals New Aspects of Crystal Growth and Elemental Composition. *Crystal Growth & Design*, **21**(10), 5521-5533. https://doi.org/10.1021/acs.cgd.1c00087

Wesson, J. A., & Ward, M. D. (2007). Pathological biomineralization of kidney stones. *Elements*, **3**(6), 415-421. https://doi.org/10.2113/GSELEMENTS.3.6.415

Wu, P., Wang, J., & Jiang, L. (2020). Bio-inspired photonic crystal patterns. *Materials Horizons*, **7**(2), 338-365. 10.1039/C9MH01389J

Yao, S., Jin, B., Liu, Z., Shao, C., Zhao, R., Wang, X., & Tang, R. (2017). Biomineralization: from material tactics to biological strategy. *Advanced Materials*, **29**(14), 1605903. https://doi.org/10.1002/adma.201605903

Zhang, G., Hirsch, A., Shmul, G., Avram, L., Elad, N., Brumfeld, V., Pinkas, I., Feldman, Y., Asher, R. B., Palmer, B. A., Kronik, L., Leiserowitz, L., Weiner, S. & Addadi, L. (2019). Guanine and 7, 8-dihydroxanthopterin reflecting crystals in the Zander fish eye: crystal locations, compositions, and structures. *Journal of the American Chemical Society*, **141**(50), 19736-19745. https://doi.org/10.1021/jacs.9b08849

Zhu, K., Chi, J., Zhang, D., Ma, B., Dong, X., Yang, J., Zhao, C. & Liu, H. (2019). Bio-inspired photonic crystals for naked eye quantification of nucleic acids. *Analyst*, **144**(18), 5413-5419. https://doi.org/10.1039/C9AN01042D

Chemical gardens

Barge, L. M., Cardoso, S. S., Cartwright, J. H., Cooper, G. J., Cronin, L., De Wit, A., Doloboff, I. J., Escribano, B., Goldstein, R. E., Haudin, F., Jones, D. E. H., Mackay, A. L., Maselko, J., Pagano, J. J., Pantaleone. J., Russell, M. J., Sainz-Díaz, C. I., Steinbock, O., Stone, D. A., Tanimoto, Y. & Thomas, N. L. (2015). From chemical gardens to chemobrionics. *Chemical Reviews*, **115**(16), 8652-8703. https://doi.org/10.1021/acs.chemrev.5b00014

Cartwright, J. H., García-Ruiz, J. M., Novella, M. L., & Otálora, F. (2002). Formation of chemical gardens. *Journal of Colloid and Interface Science*, **256**(2), 351-359. https://doi.org/10.1006/jcis.2002.8620

Getenet, M., García-Ruiz, J. M., Verdugo-Escamilla, C., & Guerra-Tschuschke, I. (2020). Mineral vesicles and chemical gardens from carbonate-rich alkaline brines of lake Magadi, Kenya. *Crystals*, **10**(6), 467. https://doi.org/10.3390/cryst10060467

Glaab, F., Kellermeier, M., Kunz, W., Morallon, E., & García-Ruiz, J. M. (2012). Formation and evolution of chemical gradients and potential differences across self-assembling inorganic membranes. *Angewandte Chemie*, **124**(18), 4393-4397. https://doi.org/10.1002/ange.201107754

Haudin, F., Cartwright, J. H., Brau, F., & De Wit, A. (2014). Spiral precipitation patterns in confined chemical gardens. *Proceedings of the National Academy of Sciences*, **111**(49), 17363-17367. https://doi.org/10.1073/pnas.1409552111

Nakouzi, E., & Steinbock, O. (2016). Self-organization in precipitation reactions far from the equilibrium. *Science Advances*, **2**(8), e1601144. doi: 10.1126/sciadv.1601144

Ritchie, C., Cooper, G. J., Song, Y. F., Streb, C., Yin, H., Parenty, A. D., MacLaren, D. A. & Cronin, L. (2009). Spontaneous assembly and real-time growth of micrometre-scale tubular structures from polyoxometalate-based inorganic solids. *Nature Chemistry*, **1**(1), 47-52. https://doi.org/10.1038/nchem.113

Stone, D. A., & Goldstein, R. E. (2004). Tubular precipitation and redox gradients on a bubbling template. *Proceedings of the National Academy of Sciences*, **101**(32), 11537-11541. https://doi.org/10.1073/pnas.0404544101

Thouvenel-Romans, S., & Steinbock, O. (2003). Oscillatory growth of silica tubes in chemical gardens. *Journal of the American Chemical Society*, **125**(14), 4338-4341. https://doi.org/10.1021/ja0298343

Structure of guests in MOF and their applications

Kapustin, I. (2018). Structure of guests in MOF and their applications (Doctoral dissertation, UC Berkeley). https://escholarship.org/uc/item/1bx6b07s

Lee, S., Kapustin, E. A., & Yaghi, O. M. (2016). Coordinative alignment of molecules in chiral metal-organic frameworks. *Science*, **353**(6301), 808-811. DOI: 10.1126/science.aaf9135

Pei, X., Bürgi, H. B., Kapustin, E. A., Liu, Y., & Yaghi, O. M. (2019). Coordinative alignment in the pores of MOFs for the structural determination of N-, S-, and P-containing organic compounds including complex chiral molecules. *Journal of the American Chemical Society*, **141**(47), 18862-18869. https://doi.org/10.1021/jacs.9b10501

Crystallization and chirality. Viedma ripening.

Baglai, I., Leeman, M., Kellogg, R. M., & Noorduin, W. L. (2019). A Viedma ripening route to an enantiopure building block for Levetiracetam and Brivaracetam. *Organic & Biomolecular Chemistry*, **17**(1), 35-38. 10.1039/C8OB02660B

Baglai, I., Leeman, M., Wurst, K., Kaptein, B., Kellogg, R. M., & Noorduin, W. L. (2018). The Strecker reaction coupled to Viedma ripening: a simple route to highly hindered enantiomerically pure amino acids. *Chemical Communications*, **54**(77), 10832-10834. https://doi.org/10.1039/C8CC06658B

Engwerda, A. H., Meekes, H., Kaptein, B., Rutjes, F. P., & Vlieg, E. (2016). Speeding up Viedma ripening. *Chemical Communications*, **52**(81), 12048-12051. 10.1039/c6cc06766b

Hananel, U., Moshe, A. B., Markovich, G., & Alivisatos, A. P. (2021). Nanocrystals as Model Systems for Studying the Interplay between Crystallization and Chirality. *Israel Journal of Chemistry*. https://doi.org/10.1002/ijch.202100058

Tarasevych, A. V., Sorochinsky, A. E., Kukhar, V. P., Toupet, L., Crassous, J., & Guillemin, J. C. (2015). Attrition-induced spontaneous chiral amplification of the γ polymorphic modification of glycine. CrystEngComm, 17(7), 1513-1517. https://doi.org/10.1039/C4CE02434F

Sairanova, N. I. & Gainullina, Y. (2021). A Chiral Stationary Phase Based on Guanine Conglomerates Obtained under Viedma Ripening Conditions. *Journal of Analytical Chemistry*, **76**(11), 1321-1326. https://doi.org/10.1134/S1061934821110125

Sivakumar, R., Askari, M. S., Woo, S., Madwar, C., Ottenwaelder, X., Bohle, D. S., & Cuccia, L. A. (2016). Homochiral crystal generation via sequential dehydration and Viedma ripening. *CrystEngComm*, **18**(23), 4277-4280. https://doi.org/10.1039/C6CE00119J

Sögütoglu, L. C., Steendam, R. R., Meekes, H., Vlieg, E., & Rutjes, F. P. (2015). Viedma ripening: a reliable crystallisation method to reach single chirality. *Chemical Society Reviews*, **44**(19), 6723-6732. 10.1039/C5CS00196J

Viedma, C. (2007). Chiral symmetry breaking and complete chiral purity by thermodynamic-kinetic feedback near equilibrium: implications for the origin of biochirality. *Astrobiology*, **7**(2), 312-319. https://doi.org/10.1089/ast.2006.0099

Viedma, C., Coquerel, G., & Cintas, P. (2015). Crystallization of chiral molecules. In *Handbook of Crystal Growth* (pp. 951-1002). Elsevier. https://doi.org/10.1016/B978-0-444-56369-9.00022-8

Viedma, C., McBride, J. M., Kahr, B., & Cintas, P. (2013). Enantiomer-Specific Oriented Attachment: Formation of Macroscopic Homochiral Crystal Aggregates from a Racemic System. *Angewandte Chemie*, **125**(40), 10739-10742. https://doi.org/10.1002/ange.201303915

Viedma, C., & Ortiz, J. E. (2021). A New Twist in Eutectic Composition: Deracemization of a Racemic Compound Amino Acid by Viedma Ripening and Temperature Fluctuation. *Israel Journal of Chemistry*. First published: 19 October 2021, https://doi.org/10.1002/ijch.202100075

Confined crystallization

Jiang, Q., & Ward, M. D. (2014). Crystallization under nanoscale confinement. Chemical Society Reviews, 43(7), 2066-2079. https://doi.org/10.1039/C3CS60234F

Lee, A. Y., Lee, I. S., Dette, S. S., Boerner, J., & Myerson, A. S. (2005). Crystallization on confined engineered surfaces: A method to control crystal size and generate different polymorphs. *Journal of the American Chemical Society*, **127**(43), 14982-14983. https://doi.org/10.1021/ja055416x

Liu, G., Müller, A. J., & Wang, D. (2021). Confined crystallization of polymers within nanopores. *Accounts of Chemical Research*, **54**(15), 3028-3038. https://doi.org/10.1021/acs.accounts.1c00242

Meldrum, F. C., & O'Shaughnessy, C. (2020). Crystallization in confinement. *Advanced Materials*, **32**(31), 2001068. https://doi.org/10.1002/adma.202001068

Piorkowska, E., Billon, N., Haudin, J. M., & Gadzinowska, K. (2005). Spherulitic structure development during crystallization in confined space II. Effect of spherulite nucleation at borders. *Journal of Applied Polymer Science*, **97**(6), 2319-2329. https://doi.org/10.1002/app.21802

Rijniers, L. A., Huinink, H. P., Pel, L., & Kopinga, K. (2005). Experimental evidence of crystallization pressure inside porous media. *Physical Review Letters*, **94**(7), 075503. https://doi.org/10.1103/PhysRevLett.94.075503

High-pressure crystallization

Abbas, N., Oswald, I. D., & Pulham, C. R. (2017). Accessing mefenamic acid form II through high-pressure recrystallisation. *Pharmaceutics*, **9**(2), 16. https://doi.org/10.3390/pharmaceutics9020016

Katrusiak, A. (2008). High-pressure crystallography. *Acta Crystallographica Section A: Foundations of Crystallography*, **64**(1), 135-148. https://doi.org/10.1107/S0108767307061181

Katrusiak, A. (2019). Lab in a DAC–high-pressure crystal chemistry in a diamond-anvil cell. *Acta Crystallographica Section B: Structural Science, Crystal Engineering and Materials*, **75**(6), 918-926. https://doi.org/10.1107/S2052520619013246

Oswald, I. D., Allan, D. R., Day, G. M., Motherwell, W. S., & Parsons, S. (2005). Realizing predicted crystal structures at extreme conditions: the low-temperature and high-pressure crystal structures of 2-chlorophenol and 4-fluorophenol. *Crystal Growth & Design*, **5**(3), 1055-1071. https://doi.org/10.1021/cg049647b

Oswald, I. D., Chataigner, I., Elphick, S., Fabbiani, F. P., Lennie, A. R., Maddaluno, J., Marshall, W. G., Prior, T. J., Pulham, C. R. & Smith, R. I. (2009). Putting pressure on elusive polymorphs and solvates. *CrystEngComm*, **11**(2), 359-366. https://doi.org/10.1039/B814471K

Oswald, I. D., & Pulham, C. R. (2008). Co-crystallisation at high pressure - An additional tool for the preparation and study of co-crystals. *CrystEngComm*, **10**(9), 1114-1116. https://doi.org/10.1039/B805591B

Zakharov, B. A., Goryainov, S. V., & Boldyreva, E. V. (2016). Unusual seeding effect in the liquid-assisted high-pressure polymorphism of chlorpropamide. *CrystEngComm*, **18**(29), 5423-5428. DOI: 10.1039/C6CE00711B

3D-printing and visualization of crystal structures

Brown, M. L., Hartling, D., Tailor, H. N., Van Wieren, K., Houghton, G. B., McGregor, I. G., Hansen, C. D. & Merbouh, N. (2019). Piecewise 3D printing of crystallographic data for post-printing construction. *CrystEngComm*, **21**(38), 5757-5766. https://doi.org/10.1039/C9CE00986H

Brown, M. L., Van Wieren, K., Tailor, H. N., Hartling, D., Jean, A., & Merbouh, N. (2018). Three-dimensional printing of ellipsoidal structures using Mercury. *CrystEngComm*, **20**(3), 271-274. https://doi.org/10.1039/C7CE01901G

Macdonald, N. P., Bunton, G. L., Park, A. Y., Breadmore, M. C., & Kilah, N. L. (2017). 3D printed micrometer-scale polymer mounts for single crystal analysis. *Analytical Chemistry*, **89**(8), 4405-4408. https://doi.org/10.1021/acs.analchem.7b00443

Moeck, P., DeStefano, P., Kaminsky, W., & Snyder, T. (2019). 3D printing in the context of Science, Technology, Engineering, and Mathematics education at the college/university level. *arXiv preprint arXiv:2001.04267*. https://arxiv.org/abs/2001.04267

Moeck, P., DeStefano, P., Kaminsky, W., & Snyder, T. (2020). 3D Printing in the Context of Science, Technology, Engineering, and Mathematics Education at the College/University Level. In: 21st Century Nanoscience–A Handbook: Public Policy, Education, and Global Trends (Volume 10, Chapter 14). ISBN 1000705382, 9781000705386

Scalfani, V. F., Williams, A. J., Tkachenko, V., Karapetyan, K., Pshenichnov, A., Hanson, R. M., Liddie, J. M. & Bara, J. E. (2016). Programmatic conversion of crystal structures into 3D printable files using Jmol. *Journal of Cheminformatics*, **8**(1), 1-8. https://doi.org/10.1186/s13321-016-0181-z

Van Wieren, K., Tailor, H. N., Scalfani, V. F., & Merbouh, N. (2017). Rapid access to multicolor three-dimensional printed chemistry and biochemistry models using visualization and three-dimensional printing software programs. *Journal of Chemical Education*, **94**(7), 964-969. https://doi.org/10.1021/acs.jchemed.6b00602

Crystals and chocolate

Fryer, P., & Pinschower, K. (2000). The materials science of chocolate. *Mrs Bulletin*, **25**(12), 25-29. DOI: https://doi.org/10.1557/mrs2000.250

van Langevelde, A., van Malssen, K., Sonneveld, E. D., Peschar, R., & Schenk, H. (1999). Crystal packing of a homologous series β′-stable triacylglycerols. *Journal of the American Oil Chemists' Society*, **76**(5), 603-609. https://doi.org/10.1007/s11746-999-0010-x

van Langevelde, A., Van Malssen, K., Peschar, R., & Schenk, H. (2001). Effect of temperature on recrystallization behavior of cocoa butter. *Journal of the American Oil Chemists' Society*, **78**(9), 919-925. https://doi.org/10.1007/s11746-001-0364-2

Van Mechelen, J. B., Peschar, R., & Schenk, H. (2006). Structures of mono-unsaturated triacylglycerols. II. The β2 polymorph. *Acta Crystallographica Section B: Structural Science*, **62**(6), 1131-1138. https://doi.org/10.1107/S0108768106037086

van Mechelen, J. B., Peschar, R., & Schenk, H. (2015). The crystal structures of the β1 and β2 polymorphs of mono-unsaturated triacylglycerols and cocoa butter determined from high resolution powder diffraction data. In *Tenth European Powder Diffraction Conference* (pp. 599-604). Oldenbourg Wissenschaftsverlag. https://doi.org/10.1524/9783486992540-094

Peschar, R., Pop, M. M., De Ridder, D. J., van Mechelen, J. B., Driessen, R. A., & Schenk, H. (2004). Crystal structures of 1, 3-distearoyl-2-oleoylglycerol and cocoa butter in the β (V) phase reveal the driving force behind the occurrence of fat bloom on chocolate. *The Journal of Physical Chemistry B*, **108**(40), 15450-15453. https://doi.org/10.1021/jp046723c

Pirouzian, H. R., Konar, N., Palabiyik, I., Oba, S., & Toker, O. S. (2020). Pre-crystallization process in chocolate: Mechanism, importance and novel aspects. *Food chemistry*, **321**, 126718. https://doi.org/10.1016/j.foodchem.2020.126718

Schenk, H., & Peschar, R. (2004). Understanding the structure of chocolate. *Radiation Physics and Chemistry*, **71**(3-4), 829-835. https://doi.org/10.1016/j.radphyschem.2004.04.105

Schenk, H., Peschar, R., Pop, M. M., De Ridder, D. J., van Mechelen, J. B., & Driessen, R. A. (2005). Crystal Structure of Chocolate from Powder Diffraction Data. *Acta Crystallographica A. Foundations and Advances*, **61**, C280-C280. http://iucr2005.iucr.org/pdf/2331.pdf

Stapley, A. G., Tewkesbury, H., & Fryer, P. J. (1999). The effects of shear and temperature history on the crystallization of chocolate. *Journal of the American Oil Chemists' Society*, **76**(6), 677-685. https://doi.org/10.1007/s11746-999-0159-3

Mechanochemical crystallization

Bokhonov, B. B., & Boldyrev, V. V. (1989). Mechanochemical synthesis of icosahedral phases in Mg-Zn-Al and Mg-Cu-Al alloys. *Reactivity of Solids*, **7**(2), 167-172. https://doi.org/10.1016/0168-7336(89)80026-9

Bokhonov, B., & Konstanchuk, I. (1991). Synthesis and process characterization of mechanically alloyed icosahedral phase Mg-Zn-Al. *Journal of Materials Science*, **26**(5), 1409-1411. https://doi.org/10.1007/BF00544486

Bokhonov, B., Konstanchuk, I., Boldyrev, V., & Ivanov, E. (1993). HRTEM study of milling induced phase transition and quasicrystalline formation in $Mg_{32}(Al, Zn)_{49}$ cubic Frank-Kasper phase. *Journal of Non-Crystalline Solids*, **153**, 606-610. https://doi.org/10.1016/0022-3093(93)90424-V

Bokhonov, B., Konstanchuk, I., Ivanov, E., & Boldyrev, V. (1992). Stage formation of quasi-crystals during mechanical treatment of the cubic Frank-Kasper phase $Mg_{32}(Zn, Al)_{49}$. *Journal of Alloys and Compounds*, **187**(1), 207-214. https://doi.org/10.1016/0925-8388(92)90534-G

Ivanov, E. Y., Konstanchuk, I. G., Bokhonov, B. D., & Boldyrev, V. V. (1989). Mechanochemical synthesis of icosahedral phases in Mg-Zn-Al and Mg-Cu-Al alloys. *Reactivity of Solids*, **7**(2), 167-172. https://doi.org/10.1016/0168-7336(89)80026-9

Loya, J. D., Li, S. J., Unruh, D. K., & Hutchins, K. M. (2021). Mechanochemistry as a Tool for Crystallizing Inaccessible Solids from Viscous Liquid Components. *Crystal Growth & Design,* **22**, 1, 285–292. https://doi.org/10.1021/acs.cgd.1c00929

Temuujin, J., Okada, K., & MacKenzie, K. J. (1999). Effect of mechanochemical treatment on the crystallization behaviour of diphasic mullite gel. *Ceramics International*, **25**(1), 85-90. https://doi.org/10.1016/S0272-8842(98)00005-4

Liquid crystals

Andrienko, D. (2018). Introduction to liquid crystals. *Journal of Molecular Liquids*, **267**, 520-541. https://doi.org/10.1016/j.molliq.2018.01.175

Chandrasekhar, S. (1993). Discotic liquid crystals. A brief review. *Liquid Crystals*, **14**(1), 3-14. https://doi.org/10.1080/02678299308027301

Meyer, R. B. (1977). Ferroelectric liquid crystals; a review. *Molecular Crystals and Liquid Crystals*, **40**(1), 33-48. https://doi.org/10.1080/15421407708084469

Del Pozo, M., Sol, J. A., Schenning, A. P., & Debije, M. G. (2021). 4D Printing of Liquid Crystals: What's Right for Me? *Advanced Materials*, 2104390. https://doi.org/10.1002/adma.202104390

Priestly, E., Wojtowisz, P. & Sheng, P. (Eds.) (2012). Introduction to Liquid Crystals. Springer Science & Business Media, 353 pp. DOI: 10.1007/978-1-4684-2175-0

Shang, Y., Wang, J., Ikeda, T., & Jiang, L. (2019). Bio-inspired liquid crystal actuator materials. *Journal of Materials Chemistry C*, **7**(12), 3413-3428. https://doi.org/10.1039/C9TC00107G

Crystallization and archaeometry

Artioli, G., & Bullard, J. W. (2013). Cement hydration: the role of adsorption and crystal growth. *Crystal Research and Technology*, **48**(10), 903-918. https://doi.org/10.1002/crat.201200713

Artioli G., Cotte M., Delgado M., Rafalska-Lasocha A.: CrysAC activities towards a crystallography-based knowledge of archaeometry and conservation. Materials Structure 26, 94-96, 2019. ISSN: 1211-5894 (print) 1805-4382 (online)

Urbanová, P., Boaretto, E., & Artioli, G. (2020). The state-of-the-art of dating techniques applied to ancient mortars and binders: A review. *Radiocarbon*, **62**(3), 503-525. https://doi.org/10.1017/RDC.2020.43

Appendix 3. Educational courses given at the Chair of Solid State Chemistry of Novosibirsk State University that can be taken by students complementary to the course on Crystal Growth

General courses:

- Solid State Chemistry – for all 3rd year chemistry students, 51 hours* lectures, 51 hours practicals, includes crystallography and crystal chemistry
- Supramolecular Chemistry - for all 4th year chemistry students, 20 hours lectures

Courses of choice:

- Introduction into diffraction techniques. Basics - 17 hours lectures, 17 hours practicals
- Introduction into diffraction techniques. Advanced level - 17 hours lectures, 17 hours practicals
- Introduction into structural biology - 16 hours lectures, 16 hours practicals
- Materials for large-scale facilities - 16 hours lectures, 16 hours practicals
- Low-dimensional materials - 16 hours lectures, 16 hours practicals
- Introduction into the structural databases -12 hours lectures, 22 hours practicals
- Introduction into synchrotron and neutron techniques -32 hours lectures
- From drug substances to drug formulations - 18 hours lectures, 18 hours practicals
- Materials for energy and catalysis 16 hours lectures, 16 hours practicals
- Physico-chemical analysis in solid state chemistry and materials sciences -20 hours lectures, 12 hours laboratory training
- Introduction into materials sciences 16 hours lectures, 16 hours practicals
- High-pressure techniques 16 hours lectures, 16 hours practicals

  * - throughout the text, we refer to course hours. Homework is not taken into account

Appendix 4. Selected links to the resources related to teaching crystal growth at different levels and for different audiences available on-line

Practical aspects of crystal growth for students and general public:

1. Project "Fascinating Science" for children; Проект "Занимательная наука для школьников" https://cfc.nsu.ru/?author=3
2. Crystals; http://zircon81.narod.ru/Metodica.html
3. How to grow a single crystal: https://www.youtube.com/watch?v=cNyQ_pMGxWs
4. A project "Silicate garden" https://infourok.ru/proekt-po-himii-silikatniy-sad-1241109.html
5. Tips and tricks (techniques of crystallization): http://www.chemistryviews.org/details/education/2532131/Tips_and_Tricks_for_the_Lab_Growing_Crystals_Part_1.html ; http://www.chemistryviews.org/details/education/2538901/Tips_and_Tricks_for_the_Lab_Growing_Crystals_Part_2.html ; http://www.chemistryviews.org/details/education/2538941/Tips_and_Tricks_for_the_Lab_Growing_Crystals_Part_3.html ; http://www.chemistryviews.org/details/education/4519261/Tips_and_Tricks_for_the_Lab_Air-Sensitive_Techniques_4.html

Crystal growth & crystallography

1. Crystal gallery IUCr: https://www.iycr2014.org/participate/crystal-growing/gallery & https://www.iucr.org/education/resources
2. "The Fascination of Crystals and Symmetry" project: https://crystalsymmetry.wordpress.com/230-2/
3. Training and Educational Resources CCDC: https://www.ccdc.cam.ac.uk/Community/educationalresources/

Useful video:

General:

1. "Beautiful Chemistry" project: https://www.beautifulchemistry.net/reaction
2. Crystal Growth: https://www.youtube.com/watch?v=Nt5TA2_ABJg

Crystallization of inorganic compounds:

1. KDP crystal growth: https://www.youtube.com/watch?v=l_USYub3djY
2. Timelapse shows frost through a microscope: https://www.youtube.com/watch?v=bDPczGUovzE
3. Microscopic time-lapse of growing snowflake: https://www.youtube.com/watch?v=MCA2VmDVzEo
4. The beauty of crystal growth (crystallization of sodium acetate): https://www.youtube.com/watch?v=hibmI6aGOso
5. Hot ice (sodium acetate) beautiful science experiment: https://www.youtube.com/watch?v=BLq5NibwV5g
6. Experiment - salt crystals growing - time lapse: https://www.youtube.com/watch?v=0g7qYPZHf0A
7. Time lapse video of epsom salt crystals growing under polarized light: https://www.youtube.com/watch?v=6feLRBBtC9c
8. Envisioning Chemistry Crystallization2: https://www.youtube.com/watch?v=dyVz6h2wrEA
9. Beauty of crystallization. https://www.youtube.com/watch?v=lo0cp2uhxb0&t=43s

Crystallization of organic compounds

1. Crystallisation of Vitamin C: https://www.youtube.com/watch?v=DeJw03BLTGo
2. Erythritol Crystallization: https://www.youtube.com/watch?v=oFxz7tQY-Z8
3. Thymol crystallization: https://www.youtube.com/watch?v=lffePrl-UCU

Biological & macromolecule crystallization

1. Protein crystallography: https://www.youtube.com/watch?v=8SN1kmfhWcs
2. Understanding crystallography: part 1 from protein to crystals: https://www.youtube.com/watch?v=gLsC4wlrR2A
3. Understanding crystallography: part 2 from crystals to diamond: https://www.youtube.com/watch?v=WJKvDUo3KRk
4. Single crystal diffraction familiarization video: https://www.youtube.com/watch?v=suVNYD1nCm4
5. Faster and smaller macromolecular crystallography at DLS: https://www.youtube.com/watch?v=S3g5kXJ8ib8

6. Seeding mechanism during crystallization: https://www.youtube.com/watch?v=asLtlLKPT0k
7. David J. Haas, part 1: Cryo-cooling Protein Crystals: The First 52 Years: https://www.youtube.com/watch?v=Q6Ai4dGml9M

Metals

1. Gallium - a terminator metal https://www.youtube.com/watch?v=iPlhdzMKp6A
2. Beautiful Chemical Reactions - Metal Displacement https://www.youtube.com/watch?v=C7ZZWXI6gLs
3. Envisioning chemistry- metal displacement II https://www.youtube.com/watch?v=lT2v7JgiGSY
4. Envisioning Chemistry- Disappearing Metals https://www.youtube.com/watch?v=AcYaQyfgg8o
5. Microscopic time-lapse- see the crazy chemistry of reacting metal - short film showcase https://www.youtube.com/watch?v=RSrLwG25euc

Liquid crystals

1. What are Liquid Crystals? https://www.youtube.com/watch?v=MuWDwVHVLio
2. Crystallization of cholesterylpelargonate https://www.youtube.com/watch?v=i8Krw0xOagg
3. Birefringence and phase transition of the liquid crystal Cholesteryl Pelargonate 97% https://www.youtube.com/watch?v=9Ku0MHWt6bw

Semiconductors

1. Process of obtaining single-crystals of Si: https://youtu.be/8QKzS_w_Ko0
2. Some more about the Czochralski method: https://youtu.be/xftnhfa-Dmo
3. p- and n-conductivity and transistors: https://youtu.be/jh2z-g7GJxE
4. Wafer manufacturing process: https://youtu.be/3TOpg1niATg
5. Silicon vs Germanium. Differences between Si and Ge: https://www.youtube.com/watch?v=iR62tf4NkcA

“Chemical garden”:

1. Chemical garden https://en.wikipedia.org/wiki/Chemical_garden
2. «Chemical garden» https://elementy.ru/kartinka_dnya/224/Khimicheskiy_sad

3. The Chemical Garden - A time lapse movie
https://www.youtube.com/watch?v=z4SiKBfFRbE

4. Envisioning Chemistry- Chemical Garden
II: https://www.youtube.com/watch?v=JslO9tiBaSw

Miscelleneous:

1. Spherulite Formation: https://www.youtube.com/watch?v=9qbCyJ-nF5A
2. Envisioning Chemistry- Getting Hot (with Thermal Imaging):
https://www.youtube.com/watch?v=VSyEWg10WjM
3. Envisioning Chemistry- Precipitation IV:
https://www.youtube.com/watch?v=603k16Rx7YU
4. Crystals under microscope 2: https://www.youtube.com/watch?v=SsvJtKNrsj8
5. Timelapse of Crystals Growing: https://www.youtube.com/watch?v=eHfO8pmaXMg

YouTube channels:

1. Beauty of Science:
https://www.youtube.com/channel/UCYmL90kuJusvrpVv3B60Rsg
2. International Union of Crystallography https://www.youtube.com/user/theIUCr
3. BioXFEL https://www.youtube.com/user/BioXFEL
4. Rigaku Corporation https://www.youtube.com/c/RigakuCorporation
5. CrystalGrower https://www.youtube.com/channel/UCnftdepWN2iAt_hz8s2WhQg
6. Frank Hoffmann https://www.youtube.com/c/FrankHoffmann1000
7. Distant learnig at the Faculty of Materials Sciences of the Moscow State University;
ФНМ МГУ https://www.youtube.com/channel/UCepiCWoe2ew8ID901LL4Fkg

Simulators and games:

1. vDiffraction: A serious game about diffraction and crystals
https://www.ill.eu/users/support-labs-infrastructure/software-scientific-tools/vdiffraction/
2. EwaldSphere (simulates a 3-circle single-crystal X-ray diffractometer)
http://academic.sun.ac.za/barbour/Software.html

Virtual tours to large-scale facilities:

1. ILL https://www.ill.eu/about-the-ill/documentation/films-animations/ill-virtual-tour

2. BESSY II https://www.helmholtz-berlin.de/zentrum/aktuell/mediathek/360/panoramen_en.html#c504498 ; https://www.helmholtz-berlin.de/zentrum/aktuell/mediathek/360/index_en.html
3. «Nauka 360°»: https://360.rscf.ru/#projects
4. SuperrAIntgenlaser XFEL - Lebensbausteine im Blick - Projekt Zukunft https://www.youtube.com/watch?v=oLTL3sJqK4U
5. Der neue RAIntgenlaser European XFEL - Tunnelflug https://www.youtube.com/watch?v=p3G90p4glQA
6. FLASH - DESY's High-Speed Camera for the Nanocosmos https://www.youtube.com/watch?v=hC6TT-0gQcU

Appendix 5. Sample plans of selected lectures

5a. General introduction. Crystals from geology to biology

Different types of crystals. Relation between crystal structure and the type of chemical bonds and interactions. Examples of very large and very small crystals.

Different definitions of a crystal. How and why they changed with time. 1D, 2D, 3D crystals.

Parameters to be controlled on crystal growth:

- Chemical formula
- Polymorph
- Size
- Shape
- Perfection / defects (types, concentration, spatial distribution)

When we need to prevent crystallization.

Stable, metastable, unstable forms.

Thermodynamics vs kinetics.

Classical / non-classical nucleation and growth.

Crystallization techniques (an overview):

- From solution
- From melt
- From gas
- As a result of a chemical reaction (also a solid-state reaction)
- Electrocrystallization
- Biocrystallization
- Effects of electromagnetic fields, or light
- High-pressure crystallization
- Confined crystallization
- Crystallization in levitating drops

Crystals in physics, chemistry, planetary sciences, biology, medicine.

Crystals used as materials and materials helping to study crystals.

Crystals and food. Chocolate, Ice-cream, Honey.

5b. Defects in crystals and crystal growth

Part 1. Classification of defects in crystals.

Zero-dimensional (point defects), one-dimensional (dislocations), two-dimensional, and three-dimensional.

Equilibrium concentration of point defects, temperature dependence. Typical values of concentration of point defects.

Edge and screw dislocations.

Part 2. Sources of dislocations in grown crystal.

Growth of dislocations from a seed crystal, uneven distribution of point defects, thermal and mechanical stresses, "mistakes" of growth (for example, the meeting of two growth islands with different orientations).

Typical values of concentration of dislocations in different crystalline materials (carefully grown high purity single crystal, annealed single crystal, annealed polycrystal, cold worked metal).

Part 3. The importance of point defects for the creation of functional single-crystalline materials.

Semiconductors with p- and n-type of conductivity.

Photo- and thermoluminescent materials with impurity luminescence.

5c. Melt crystallization and solid-state growth in Nature

Part 1. Crystal growth from silicate melt in the Earth's crust

Minerals and rocks; rock texture, inter-grain boundaries, idiomorphic and xenomorphic relations of minerals.

Deep (intrusive) and superficial (effusive) magmatic rocks. Granite and basalt as representative constituents of the Earth's crust, their mean chemical and mineral composition.

P-T conditions of the generation and crystallization of silicate magmas in the Earth's crust. "Wet" and "dry" solidus of the rock: how it determines the migration of magma towards the surface.

Phase diagrams 'diopside – anorthite' and 'k-feldspar – SiO_2' as models of crystallization of silicate melt. Liquidus phase. Restite (eutectic) melt and its textural signs. The transition from idiomorphic to xenomorphic crystals during the crystallization.

The conditions of crystallization in deep magmatic chamber and erupted lava. Cooling rates, temperature gradients, oversaturation. The variation of the rate of crystal nucleation and growth depending on oversaturation. Multistage crystal growth in basaltic melts during its ascent and effusion. The resulting textures of intrusive (granite) and effusive (basalt) rocks.

Co-crystallization from eutectic melt: oriented (graphic) intergrowths of minerals -

pegmatites. The necessary conditions (over-cooling, diffusion and crystal growth rates, the presence of water) for their growth.

Part 2. Solid state growth in the Earth's crust

Exsolution.

Solid solution. Isomorphic elements. Ordering of solid solution, its crystal chemical prerequisites. Exsolution and its thermodynamic interpretation. Solvus, immiscibility range. Exsolution processes in minerals; cooling rates and the degree of separation of the product phases. Homogeneous and heterogeneous nucleation. Energetic parameters of the nucleus surface: elastic strain and surface energy. Equilibrium (chemical) and coherent solvus. Exsolution structures in feldspars.

Metamorphic reactions.

Metamorphic processes in the Earth's crust, pressure and temperature as their principal factors. Lithostatic and tectonic pressure.

Metamorphic reaction or replacement reaction; its isochemical character. Pseudomorphs. Polymorphic transition. Polymorphism in Al_2SiO_5 system.

Impact metamorphism, its P-T parameters. Popigai astrobleme. Impact diamonds – pseudomorphs after graphite. Lonsdeilite (hexagonal diamond) as a sign of impact origin of diamond. Fine structure of lonsdeilite-diamond intergrowths.

5d. High-pressure crystallization

Part 1. Crystallization with diamonds

1.1. Why crystallize at high pressures?
   1.1.1. New target products (diamonds, other artificial minerals, materials, drugs, chemicals)
   1.1.2. Understanding structures and processes (chemical engineering, chemical bonds and interactions, theory of crystal growth, structure of solutions and melts, formation and transformations of minerals in the Earth and other planets, biology-related processes)
1.2. How to crystallize at high pressures?
   1.2.1. Pressure cells (diamond anvil cells, large volume presses, capillaries)
   1.2.2. Pressure calibration
   1.2.3. Characterization of samples *in situ* (diffraction, spectroscopy, optical microscopy)
   1.2.4. Crystallization starting from gases, liquids, solids
1.3. Crystallization starting from gases (oxygen, carbon dioxide, H_2S, gas hydrates)
1.4. Crystallization starting from liquids
   1.4.1. Pure liquids
   1.4.2. Solutions of solids in liquids

1.4.3. Obtaining phases metastable at ambient pressures and their recovery
1.4.4. Thermodynamics
1.4.5. Kinetics
1.5.Crystallization starting from solids
1.5.1. Solids immersed in a fluid (recrystallization)
1.5.2. Solid mixtures (crystallization on synthesis)
1.5.3. Thermodynamics
1.5.4. Kinetics

Part 2. Crystallization of diamonds

2.1. Crystallization of diamonds as target materials

2.1.1. Nanodiamonds

2.1.2. Large single crystals

2.1.3. Doped diamonds for special applications

2.2. Crystallization of diamonds, in order to understand the natural processes of their growth. Differences with procedures used in materials sciences.

5e. Biocrystallization

Biopolymers, DNA, RNA, proteins, peptides, nucleotides.

Peptide bond.

Methods of obtaining biopolymers.

Molecular mass and the number of the amino acid residues. Examples of an insulin monomer, lysozyme, influenza virus glycoprotein, eukaryotic small ribosomal subunit (40S). The sizes of hemoglobin and erythrocyte size.

Requirements to a solution of a biopolymer suitable for crystallization.

A general overview of the methods of structure determination of biopolymers with analysis of the Protein Database (PDB) statistics on the number of structures defined by each method.

Alpha Fold2.

The main stages of the crystallization of the biopolymers.

Concepts: the region of the undersaturation, solubility curve, metastable zone, nucleation zone, precipitation zone, the supersaturation region, phase diagrams for the systems where supersaturation is achieved by using an antisolvent.

Methods of the crystallization of biopolymers and different routes of reaching nucleation: Vapor-diffusion method (hanging drop, sitting drop, sandwich drop), batch experiments, dialysis, Free interface diffusion method. The method of counter-diffusion.

«Streek seeding».

Crystallization of membrane proteins.

Equipment for preparing protein solutions, micro- and nano- droplets, crystal growth at a constant desirable temperature.

Chip crystallization.

Appendix 6. Descriptions of selected laboratory exercises

6a. Crystallization of alums

Main goal – crystal growth of potassium alum crystals

Approximate time limit – 2 academic hours plus 4-6 weeks for crystal growth

Tasks

1. Learning how to grow seed crystals
2. Preparation of saturated solution of potassium alums
3. Crystal growth of potassium alums from saturated solution by slow evaporation using seed crystals
4. Description of crystal habit and comparison with the crystals of other substances
5. Preparation of a spherical crystal and observation of faceting in isothermal conditions

Substances and materials

1. Alums
2. Seed crystals of alums
3. Distillated water

Equipment

1. Laboratory coat, goggles, gloves, heating plate, glass beakers (100 ml), glass funnels, filter paper, Petri dish, spatulas, toothpick, thread, sandpaper, microscope.

Description of lab work

Preparation of seed crystals (carried out two days before the class, can be done by assistant)

A solution of potassium alum in distilled water is prepared in the ratio of ~ 5 g per 50 ml with slight heating (~ 50 °C). If needed, some extra amount of the substance is added until an insoluble precipitate is formed. Part of the solution is filtered into a Petri dish and is left to crystallize the seeds (this process may take from several hours to 1 day). It is preferable to use a Petri dish as a crystallization container to increase the evaporation area and hence crystallization rate. In addition, due to the low side wall of the dish, the extraction of crystals from the mother liquor is easier than from a beaker. Once crystallized, the seed crystals are removed from the solution and dried on filter paper.

1. Preparation of saturated solution of potassium alums

Alums are dissolved in 1 L of distillated water until the insoluble precipitate is formed. The solution is then stirred for 15 min and then filtered into a clean glass vessel. This solution will be used immediately, to start crystallization. This same solution will be also needed over the next 4-6 weeks to top-up the mother liquor in the crystallization vessels (see below). The

following concepts are discussed in relation to this laboratory exercise: seed, saturated solution, faceting, habit.

2. Crystal growth

To obtain the best defect-free octahedral crystals, it is necessary to choose the most transparent and well-faceted seed crystals, without internal defects or inclusions (this can be achieved using an optical microscope). The selected crystal is fixed on a thread tied to a stick (toothpick, pencil, etc.) and placed into a saturated solution of potassium alum (Fig. S1a). The container for crystallization is covered with filter paper and left to grow a larger crystal. It is necessary to periodically (once every 3-4 days) check the growth of the crystal to avoid pollution of its surface with small crystals. The appearance of additional seed crystals on the thread and at the bottom of the crystallization container must also be avoided. If necessary, the thread (or the crystal surface) should be cleaned mechanically or using a hot tap water jet (without harming the target crystal). It is also necessary to check the level of the mother liquor in the container and, if it has decreased due to evaporation, top-up the beaker from the previously prepared saturated solution of potassium alum (from Step 1). After extraction from the solution and drying (Fig. 2a, Fig. S1b,c), the crystals must be stored in a sealed container to prevent dehydration, since alum crystallizes as a crystal hydrate.

3. Cutting of a spherical crystal and its growth

A large crystal grown at Step 2 is evenly sanded with sandpaper until it becomes spherical. The spherical crystal is suspended in a saturated solution of potassium alum under isothermal conditions to observe the growth figures of the faces on the surface of the originally spherical sample (2-4 weeks).

Finishing the lab work: wash all the glassware, discuss the obtained results.

6b. Crystallization of L-ascorbic acid (vitamin C) - L-serine co-crystals by slow evaporation

Main goal – crystal growth of co-crystals of L-ascorbic acid with L-serine.

Approximate time limit: 2 academic hours

Tasks:

1. Learning how to grow multicomponent single crystals by slow evaporation from droplets
2. Observing stages of nucleation and crystal growth
3. Practicing the manipulations with seed crystals
4. Preserving and storing co-crystals for further unit cell determination by single crystal X-ray diffraction

Substances and materials:

1. L-serine (105 mg)
2. L-ascorbic acid (176 mg)
3. Distilled water

Equipment:

Laboratory coat, goggles, gloves, plastic test tubes, slide glasses, Parafilm® M, syringes (1 ml) with needles, cotton wool or filter nozzles for a syringe with a pore diameter of 0.45 microns or 0.22 microns, pipette (0.1-1 ml), crystal manipulation kit, cryo-oil, marker.

Description of lab work:

Dissolve 105 mg of L-serine and 176 mg of L-ascorbic acid in 600 ml of water in plastic test tubes. Dissolution takes 10-15 minutes. Cover a glass microscope slide with Parafilm® M, so that part of the glass remains uncovered. The aqueous solution is filtered with a syringe through cotton wool or filter nozzle into a new plastic test tube. A droplet of 50 µl is placed on the glass surface and 3 bigger droplets of ca. 100 µl, 150 µl and 300 µl are put on the surface covered by Parafilm® M. Attention should be paid to the droplet shape. When placed on the glass surface the drop tends to lose its shape. Instead, when a drop is placed on the hydrophobic Parafilm® M surface, the drops maintain an arched shape. The small drop of 50 µl on the glass is used to obtain crystal nuclei that will be taken to grow bigger crystals in drops places on Parafilm® M (see Fig. S2). The following processes and concepts are discussed: solubility curve for a two-component system, change in the concentration of a dissolved compound during solvent evaporation, "metastable zone", nucleation, supersaturation, spontaneous crystallization, co-crystal.

After approximately 40 min from the beginning of the experiment, crystal nuclei from the droplet on the glass surface are transferred using a metal needle into all 3 drops of different volumes (100, 150, 300 µl) located on the Parafilm® M (see Fig. S2). Crystals should begin to grow in the 100 µl droplet, and dissolve in the larger droplets if the humidity is high. Observe the growth of crystals in the 100 µl drop directly through a microscope. After a while, put the nuclei crystals again into drops of 150 µl and 300 µl again. Observe crystal growth or dissolution of the nuclei crystals again. As soon as it is possible to grow crystals larger than 0.2 mm, the crystals are extracted from the drop. Select and preserve the single crystals in cryo-oil for further testing on a single crystal X-ray diffractometer.

Finishing the lab work: wash all the glassware, turn off all equipment, discuss the experience gained, main points of theory and the obtained results.

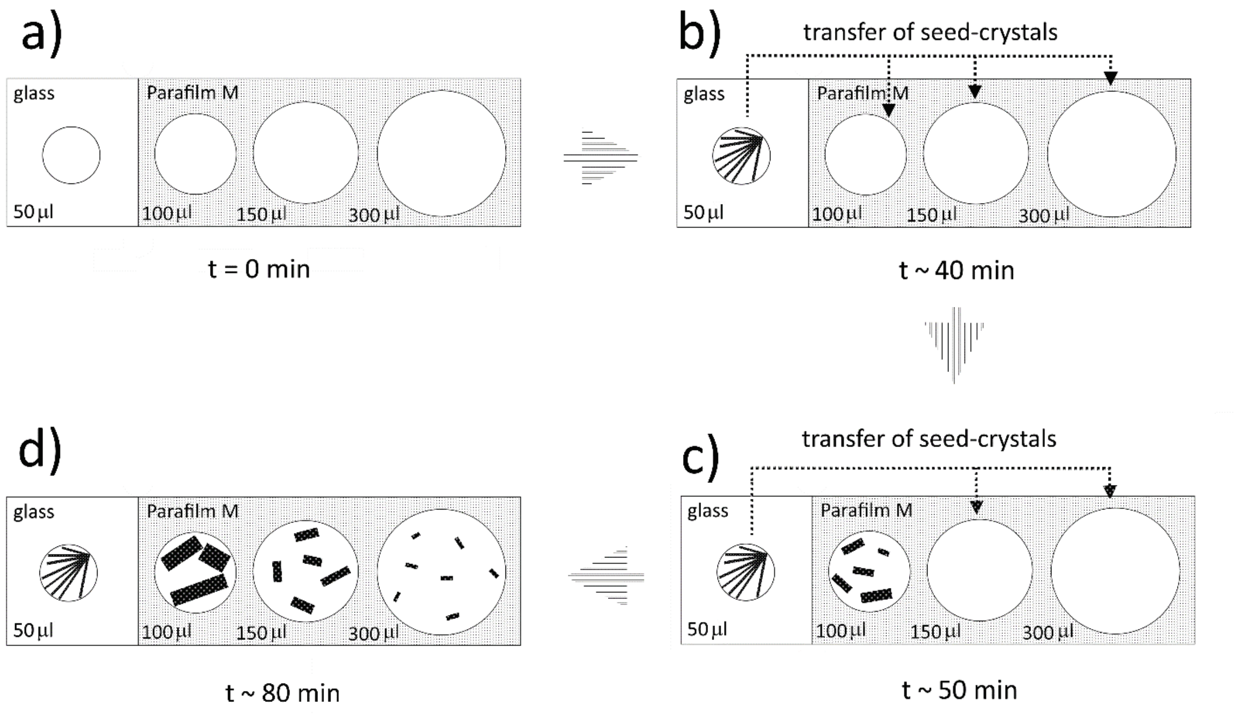

Figure S2 Scheme for the lab work 6b. a) Slide glass partly covered by Parafilm® M with 4 droplets (50 µl, 100 µl, 150 µl and 300 µl); b) Growth of seed-crystals in droplet of 50 µl and their manual transfer to other drops; c) Start of co-crystal growth in the drop of 100 µl and additional transfer of seed-crystals to the 150 µl and 300 µl drops; d) Growth of co-crystals in all drops.

6c. Lysozyme

Main goal – to get some experience with the crystallization of biopolymers

Approximate time limit: 2 academic hours

Tasks:

1. Learn about different crystallization techniques of biopolymers
2. Learn the hanging drop technique
3. Examine protein crystals in the drops under a microscope in non-polarized and polarized light

Substances and materials:

1. Hen egg-white lysozyme
2. Distilled water
3. 4 M stock solutions of NaCl
4. 1 M NaAc/HAc buffer pH = 4.0 (NaAc – sodium acetate, HAc – acetic acid)

Equipment:

Laboratory coat, goggles, gloves, 24-well hanging drop crystallization plates, P10 and P1000-type micropipettes with tips, 12 siliconized glass slides, 15 ml falcon tubes, racks for 15 ml

falcon tubes, 1.5 ml Eppendorf tubes, racks for Eppendorf tubes, pH meter, balances, laboratory fridge, microscope with polarizer and analyzer, WACKER® SILICON PASTE P4 or analog.

Description of lab work

Procedures carried out by an assistant before the class starts. It is also possible to have this work performed by the most advanced students:

15 mg of lysozyme is placed into a 1.5 ml Eppendorf tube, and dissolved in 485 μl of distilled water, thus making a 30 mg/ml lysozyme solution. Lysozyme solutions with concentrations of 50 mg/ml and 70 mg/ml should be also prepared. A 1 M NaAc/Hac stock buffer with pH = 4 is prepared by slowly adding 1 M NaAc solution to a 1 M Hac solution while stirring to reach pH = 4, ensuring that the pH is controlled during this process. The 4 M stock solutions of NaCl are prepared by dissolving in distilled water the corresponding amount of NaCl in a 500 ml volumetric flask. The lysozyme solutions are stored in the fridge.

Work done by students:

Students are divided into pairs and calculate the required volumes of 4 M NaCl stock solutions and 1 M NaAc/Hac buffer to get 2 ml precipitant solutions (i.e. NaCl in buffer solution) with NaCl concentrations of 1.1M, 1.2M, 1.3M, and 1.4M. A teacher checks the calculated ratios and then each student prepares the 2 ml solutions using P1000-type micropipettes. The teacher shows how to grease the edges of the crystallization vessel, and each student repeats this procedure.

A teacher and the students then fill the wells of a 24-well crystallization plate with the antisolvent solutions (500 μl), so that wells A1-A3 are filled with the 1.1 M NaCl solution, wells B1-B3 are filled with 1.2 M NaCl solution, wells C1-C3 are filled with 1.3 M NaCl solution, and wells D1-D3 are filled with the 1.4 M NaCl solution (Fig. S3). Each time before changing the antisolvent solution, the nozzle of the pipette must be changed.

A 2 μl droplet of the 30 mg/ml lysozyme solution is then put on the siliconized glass using a P10-type micropipette; the tip of the micropipette is removed afterwards (Fig.S4a). After that, 2 μl of the antisolvent solution from well A1 are added to the lysozyme droplet (Fig.S4b). The glass with a 4μl droplet is turned over and pasted above the antisolvent solution placed in the A1 well using grease (Fig.S4c). This is first demonstrated by a teacher, and then reproduced by the students. As a result, droplets of the 15 mg/ml lysozyme (initial concentration of the 30 mg/ml twice diluted) solution hang above wells A1, B1, C1, D1 (Fig.S4b). The same sequence is carried out with lysozyme solutions of 50 and 70 mg/ml and then placed above A2-D2 and A3-D3 wells, respectively (Fig. S3).

Above these antisolvent solutions the lysozyme crystals grow in approximately 40 mins. Students should be warned that the vapor diffusion process does not occur in such a short time and crystallization is due to the amount of precipitator solution that was added to the

drop. In the case of other proteins (not lysozyme) crystallization experiment takes from several days to weeks.

The crystals are inspected under an optical microscope in non-polarized and polarized light. The number and quality of crystals obtained under different crystallization conditions are compared and discussed.

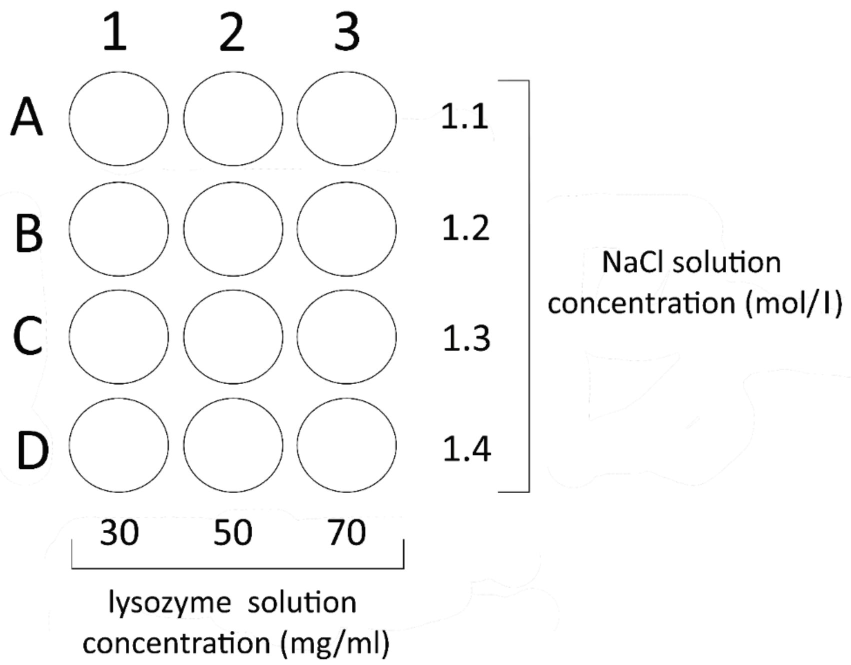

Fig. S3 Scheme of filling of a 24-well crystallization plate with a series of solutions

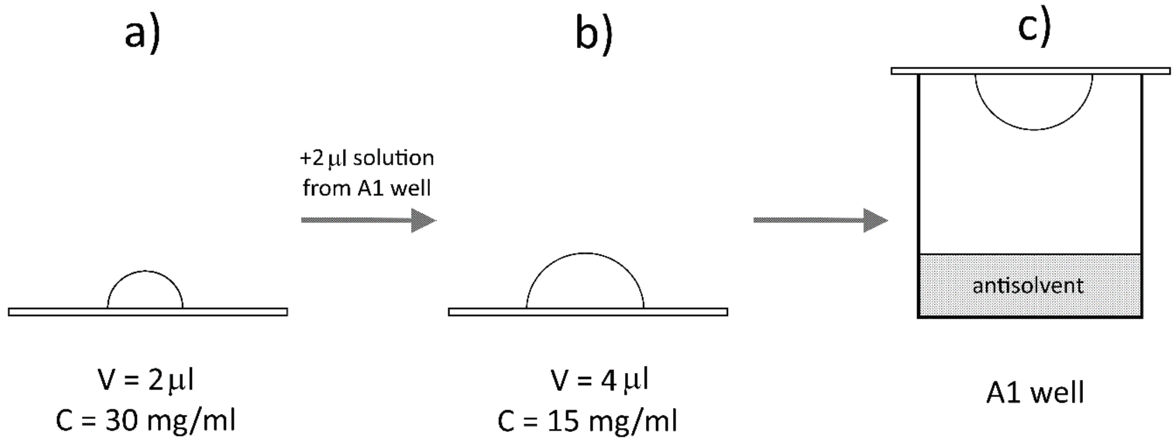

Fig. S4 The sequence of procedures to prepare a hanging drop crystallization set-up. a) Drop of lysozyme solution on siliconized glass; b) Twice diluted drop of lysozyme solution after addition of 2 µl antisolvent solution from A1 well; c) Hanging drop above antisolvent solution (A1 well)

Appendix 7. Samples of crystals grown by students

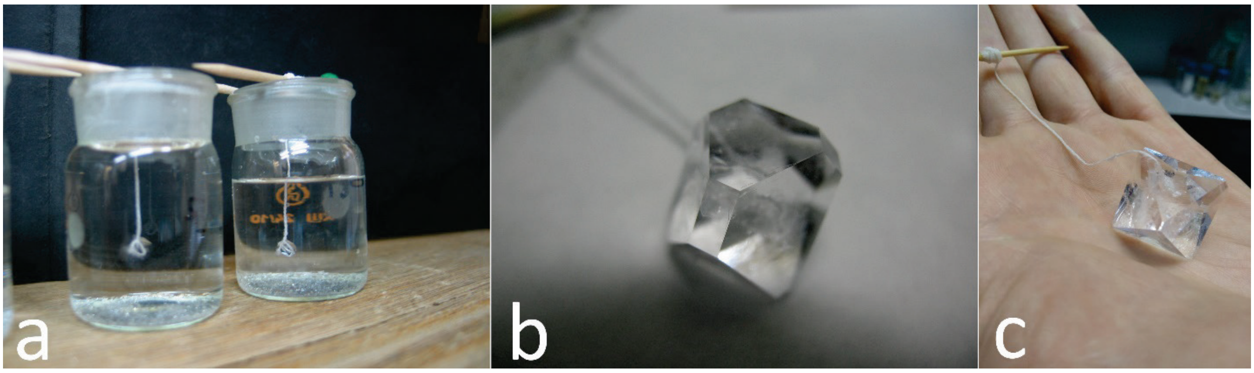

Figure S1. Potassium alum crystals grown by slow evaporation using seed crystals

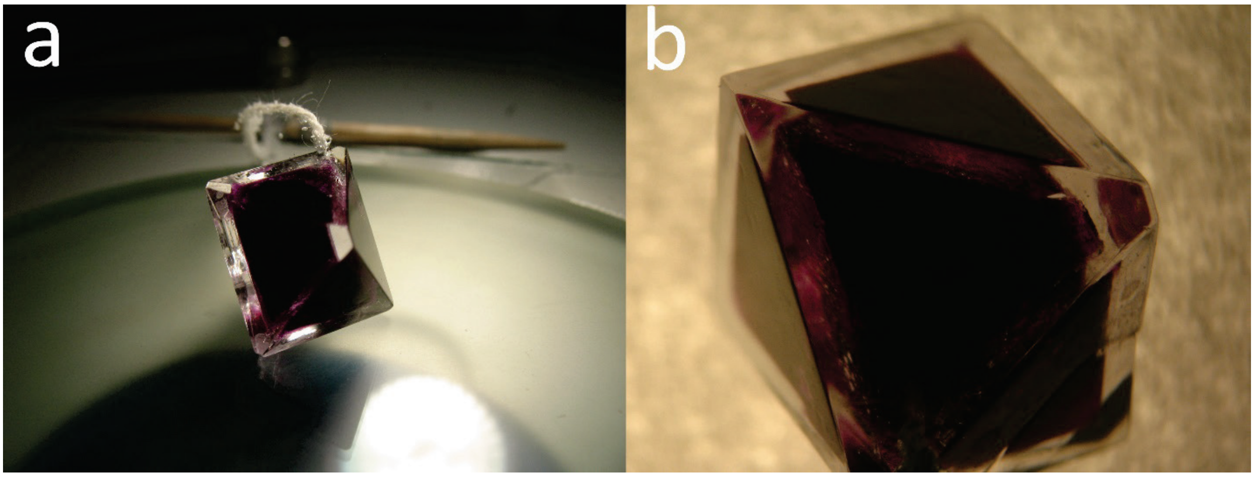

Figure S5. Growth of layered alum crystals. Chrome alum core (inside) covered by potassium alum shell (outside).

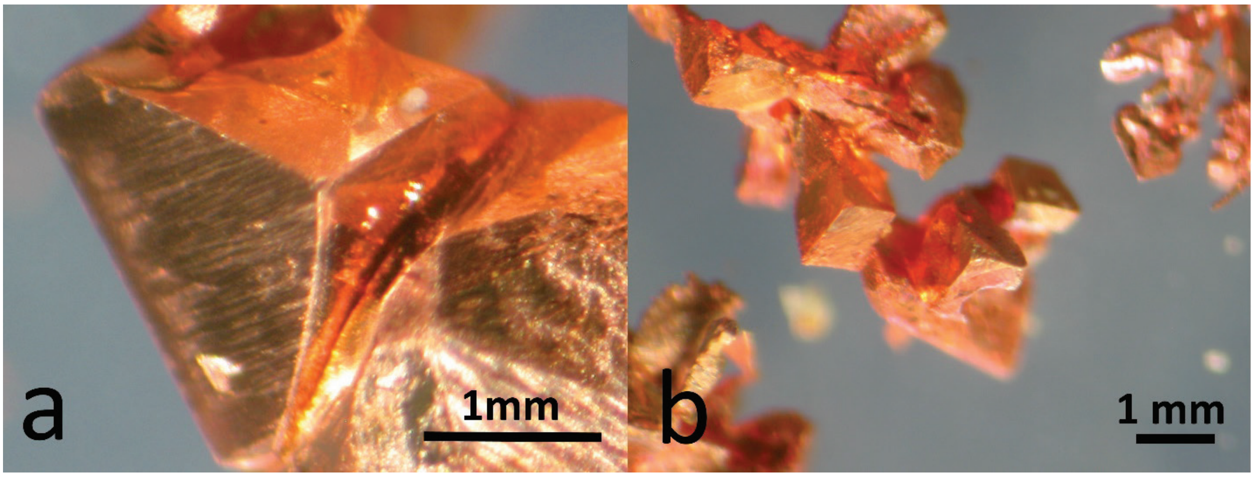

Figure S6. Copper crystals grown by redox reaction between $CuSO_4$ and Fe

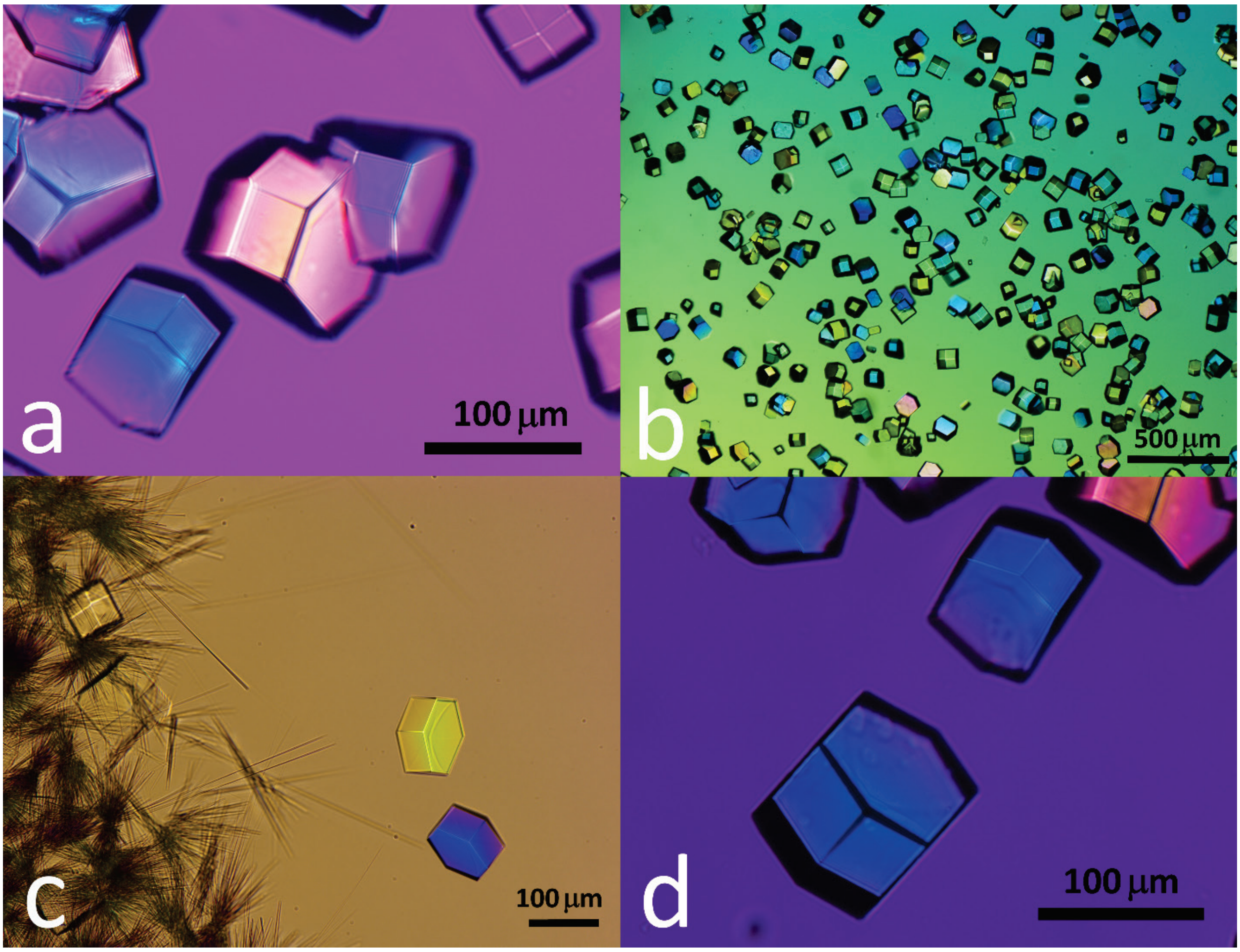

Figure S7. Lysozyme crystals under polarized light crystallized by hanging-drop vapor diffusion antisolvent technique.

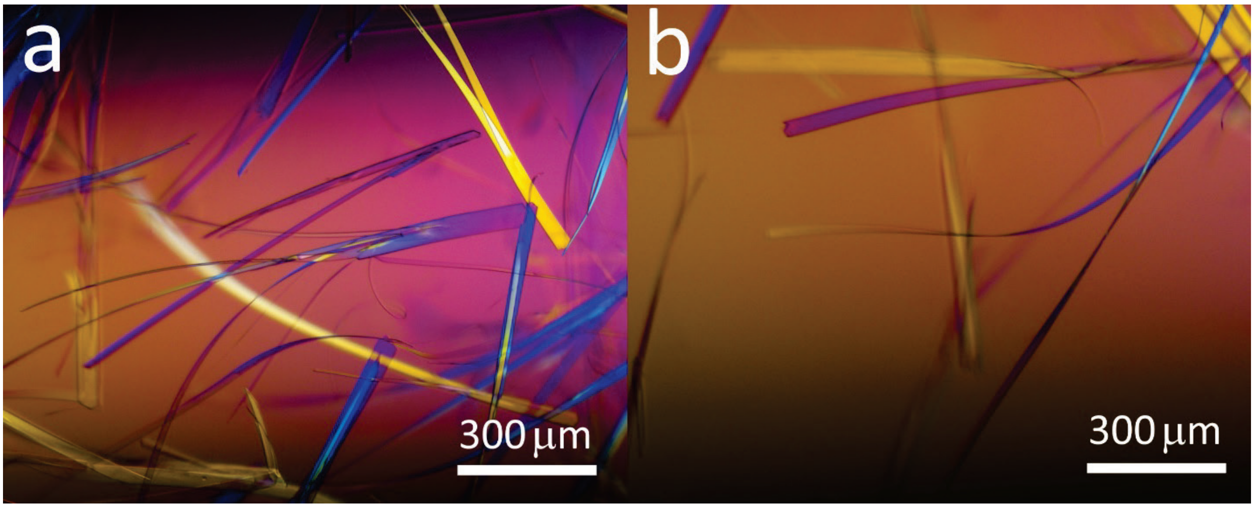

Figure S8. L-isoleucine single crystals having plastic mechanical properties were grown inside glass capillary by layering diffuse antisolvent crystallization. Optical microscopy under polarized light.

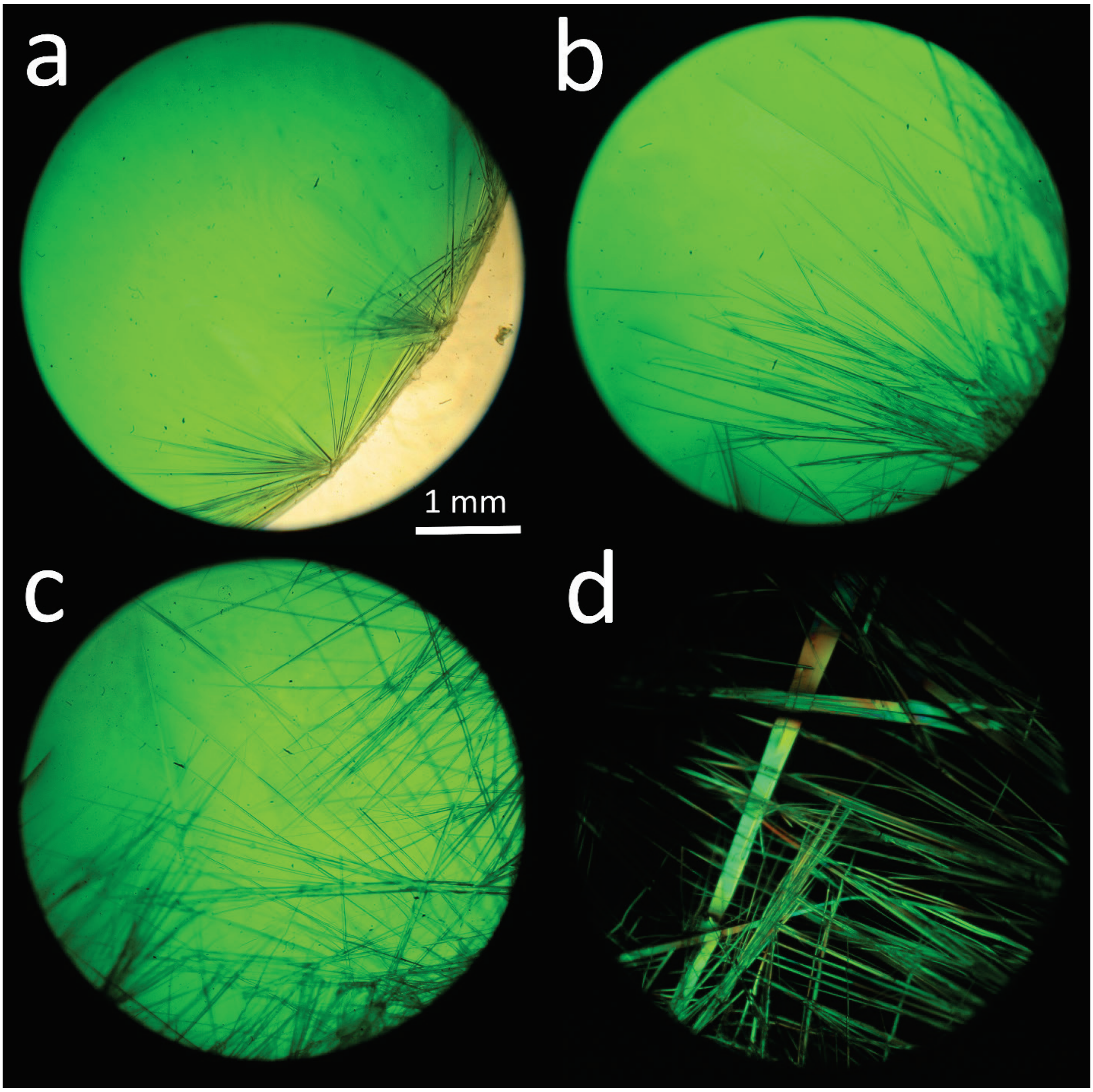

Figure S9. Growth of copper chloride dihydrate at different stages (slow evaporation crystallization from drop). Image (d) obtained using polarized light.

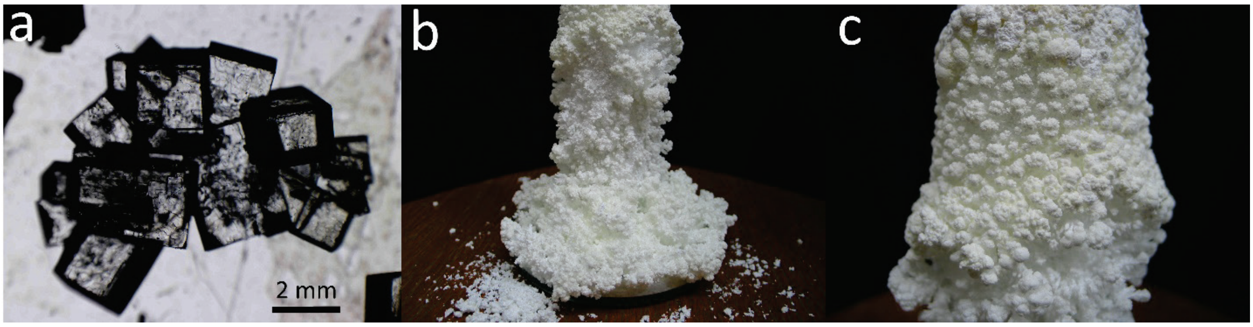

Figure S10. Sodium chloride crystals obtained by slow evaporation crystallization. a) Standard growth of cubic crystals from pure water solution and b) dendric growth from water solution with addition of $K_4[Fe(CN)_6]$

Appendix 8. List of equipment used when teaching the course

The lectures do not require any special equipment. In principle, even a chalk and a black-board would be sufficient. We use a computer and a projector for lectures, internet connection and one of the numerous programs that became so popular during the Covid restrictions, that make it possible to give and receive lectures also in a hybrid or a completely remote mode.

The laboratory exercises do not require sophisticated equipment for the basic part 1, and use the equipment of the research laboratories for part 2.

Laboratory exercises are carried out in a special room (18 m^2, laboratory tables, scales, fridge, distiller, fume hood, microscopes, individual protection means (laboratory coats, goggles, gloves), reagent storage cabinet, heating plates, ultrasonic bath, glass cups, glass funnels, filter paper, Petri dish, spatulas, toothpick, thread, sandpaper, filter nozzles for a syringe (pore diameter of 0.45 microns or 0.22 microns), pipettes, crystal manipulation kit, cryo-oil, markers.

Crystallization mushroom (Triana Sci&Tech®) (Garcia-Ruiz et al., 2002)

24-well plate (Molecular dimensions®)

Glass tubes for layering diffuse antisolvent crystallization

For advanced level experiments we use:

– A'Discoverer-1500' DIA-type apparatus with a nominal force of 1500 tons at the Sobolev Institute of Geology and Mineralogy SB RAS in Novosibirsk, Russia. Eight "Fujilloy N-05" 26 mm tungsten carbide cubes with 12 mm truncation are used as inner-stage anvils. Pyrophyllite gaskets, 4.0 mm in both width and thickness, are used to seal the compressed volume and support the anvil flanks. Parameters of the experiments: pressure up to 6 GPa, temperature up to 1500 ºC.

– Precision heating furnace (±0.1 ºC, Eurotherm 2604, RIF, BS) for solid-phase synthesis, , spontaneous crystallization on a platinum loop and crystal growth by the modified Czochralski technique (top-seeded solution growth), mechanisms for rotation and translational movement of grown crystals. Operating temperatures up to 1150 ºC (Sobolev Institute of Geology and Mineralogy SB RAS in Novosibirsk, Russia).

– Muffle furnace THERMOCERAMICS-1600 with operating temperatures up to 1600 ºC for solid phase synthesis (Sobolev Institute of Geology and Mineralogy SB RAS in Novosibirsk, Russia).

- Diamond anvil cells of different types.

- Spray-drying (Mini Spray Dryer Buchi B-290)

- A laboratory-scale freeze dryer (one processing shelf, 250 × 350 mm, with a temperature range of –30 to +80 C) (NIIC SB RAS, Russia) described in (Ogienko, A. G., Bogdanova, E. G., Trofimov, N. A., Myz, S. A., Ogienko, A. A., Kolesov, B. A., Yunoshev, A. S., Zubikov, N. V., Manakov, A. Yu., Boldyrev, V. V., & Boldyreva, E. V. (2017). Large porous particles for respiratory drug delivery. Glycine-based formulations. European Journal of Pharmaceutical Sciences, 110, 148-156)

- Mosquito Xtal3 robot for creating protein crystallization drop set

- Vibrational ball mill (Narva®, DDR)

- Vibrational ball mill Retzsch
- Home-made model devices for different types of mechanical treatment (see Tumanov, I. A., Achkasov, A. F., Boldyreva, E. V., & Boldyrev, V. V. (2011). Following the products of mechanochemical synthesis step by step. CrystEngComm, 13(7), 2213-2216; Losev E., Arkhipov S., Kolybalov D., Mineev A., Ogienko A., Boldyreva E., Boldyrev V. Substituting Steel for a Polymer in a Jar for Ball Milling does Matter, CrystEngComm. 2022.N9. P.1700-1703. DOI: 10.1039/D1CE01703A)
